# Supplementary material for: Efficacy and Safety of Epidermal Growth Factor Receptor (EGFR)-Tyrosine Kinase Inhibitor Combination Therapy as First-Line Treatment for Patients with Advanced EGFR-Mutated, Non-Small Cell Lung Cancer: A Systematic Review and Bayesian Network Meta-Analysis
Source: Cancers (Basel). 2022 Oct 6;14(19):4894. doi: 10.3390/cancers14194894 (PMC9563574; doi:10.3390/cancers14194894)
Supplement: Supplementary file 1 [file cancers-14-04894-s001.zip › cancers-1915482-supplementary.pdf]

Efficacy and Safety of Epidermal Growth Factor Receptor (EGFR) Tyrosine Kinase Inhibitor Combination Therapy as First-line Treatments for Patients with Advanced EGFR-Mutated, Non-Small cell Lung Cancer: A Systematic Review and Bayesian Network Meta-Analysis

Jianchao Xue [1,†], Bowen Li [1,†], Yadong Wang [1], Zhicheng Huang [1], Xinyu Liu [1], Chao Guo [1], Zhibo Zheng [1], Naixin Liang [1,*], Xiuning Le [3,*] and Shanqing Li [1,*]

Supplementary materials

Content

Table S1: Checklist of the PRISMA extension for network meta-analysis 3

Table S2: Retrieval strategy for the network meta-analysis in MEDLINE 9

Figure S1. Assessment of risk of bias by version 2 of the Cochrane tool for assessing the risk of bias in randomized trial (RoB2) 10

Figure S2. Forest plots of one treatment's comparison with other treatments 11

Figure S3. Diagrams showing the probability of ranks for each treatment. 14

Figure S4. The network plot and forest plot of interruption rate of TKI owing to AEs of different treatments. 15

Figure S5. The network plot and forest plot of the incidence of developing T790M mutation in patients developing the first generation TKIs resistance of different treatments. 15

Figure S6. Network plots and ranking diagrams in subgroups. 16

Figure S7. Heterogeneity assessing of the studies enrolled. 24

Figure S8. Trace plots and Brooks-Gelman-Rubin plots. 26

Figure S9. Brooks-Gelman-Rubin plots. 28

Figure S10. Forest plots of objective response rate (ORR) of afatinib plus cetuximab compared that of afatinib monotherapy. 30

Table S1: Checklist of the PRISMA extension for network meta-analysis

Section/Topic	Item #	Checklist Item	Reported on Page #
**TITLE**			
Title	1	Identify the report as a systematic review *incorporating a network meta-analysis (or related form of meta-analysis).*	***1***
**ABSTRACT**			
Structured summary	2	Provide a structured summary including, as applicable: **Background:** main objectives **Methods:** data sources; study eligibility criteria, participants, and interventions; study appraisal; and *synthesis methods, such as network meta-analysis.* **Results:** number of studies and participants identified; summary estimates with corresponding confidence/credible intervals; *treatment rankings may also be discussed. Authors may choose to summarize pairwise comparisons against a chosen treatment included in their analyses for brevity.* **Discussion/Conclusions:** limitations; conclusions and implications of findings. **Other:** primary source of funding; systematic review registration number with registry name.	***1***
**INTRODUCTION**			
Rationale	3	Describe the rationale for the review in the context of what is already known*, including mention of why a network meta-analysis has been conducted.*_	**2**
Objectives	4	Provide an explicit statement of questions being addressed, with reference to participants,	**2**

		interventions, comparisons, outcomes, and study design (PICOS).	
**METHODS**			
Protocol and registration	5	Indicate whether a review protocol exists and if and where it can be accessed (e.g., Web address); and, if available, provide registration information, including registration number.	***2***
Eligibility criteria	6	Specify study characteristics (e.g., PICOS, length of follow-up) and report characteristics (e.g., years considered, language, publication status) used as criteria for eligibility, giving rationale. *Clearly describe eligible treatments included in the treatment network, and note whether any have been clustered or merged into the same node (with justification).*_	***2~3***
Information sources	7	Describe all information sources (e.g., databases with dates of coverage, contact with study authors to identify additional studies) in the search and date last searched.	***2~3***
Search	8	Present full electronic search strategy for at least one database, including any limits used, such that it could be repeated.	***Supplementary Materials page 9***
Study selection	9	State the process for selecting studies (i.e., screening, eligibility, included in systematic review, and, if applicable, included in the meta-analysis).	***2~3***
Data collection process	10	Describe method of data extraction from reports (e.g., piloted forms, independently, in duplicate) and any processes for obtaining and confirming data from investigators.	***3***
Data items	11	List and define all variables for which data were sought (e.g., PICOS, funding sources) and any assumptions and simplifications made.	***2~3***
**Geometry of the**	**S1**	Describe methods used to explore the	***3~4***

**network**		geometry of the treatment network under study and potential biases related to it. This should include how the evidence base has been graphically summarized for presentation, and what characteristics were compiled and used to describe the evidence base to readers.	
Risk of bias within individual studies	12	Describe methods used for assessing risk of bias of individual studies (including specification of whether this was done at the study or outcome level), and how this information is to be used in any data synthesis.	***3***
Summary measures	13	State the principal summary measures (e.g., risk ratio, difference in means). *Also describe the use of additional summary measures assessed, such as treatment rankings and surface under the cumulative ranking curve (SUCRA) values, as well as modified approaches used to present summary findings from meta-analyses.*	***3***
Planned methods of analysis	14	Describe the methods of handling data and combining results of studies for each network meta-analysis. This should include, but not be limited to:<br>• *Handling of multi-arm trials;*<br>• *Selection of variance structure;*<br>• *Selection of prior distributions in Bayesian analyses; and*<br>• *Assessment of model fit.*	***3~4***
**Assessment of Inconsistency**	**S2**	Describe the statistical methods used to evaluate the agreement of direct and indirect evidence in the treatment network(s) studied. Describe efforts taken to address its presence when found.	***3~4***
Risk of bias across studies	15	Specify any assessment of risk of bias that may affect the cumulative evidence (e.g., publication bias, selective reporting within studies).	***3~4***

Additional analyses	16	Describe methods of additional analyses if done, indicating which were pre-specified. This may include, but not be limited to, the following:<br>• Sensitivity or subgroup analyses;<br>• Meta-regression analyses;<br>• *Alternative formulations of the treatment network; and*<br>• *Use of alternative prior distributions for Bayesian analyses (if applicable).*_	***3~4***
**RESULTS†**			
Study selection	17	Give numbers of studies screened, assessed for eligibility, and included in the review, with reasons for exclusions at each stage, ideally with a flow diagram.	***4***
**Presentation of network structure**	**S3**	Provide a network graph of the included studies to enable visualization of the geometry of the treatment network.	***4~5***
**Summary of network geometry**	**S4**	Provide a brief overview of characteristics of the treatment network. This may include commentary on the abundance of trials and randomized patients for the different interventions and pairwise comparisons in the network, gaps of evidence in the treatment network, and potential biases reflected by the network structure.	***4~6***
Study characteristics	18	For each study, present characteristics for which data were extracted (e.g., study size, PICOS, follow-up period) and provide the citations.	***17~18***
Risk of bias within studies	19	Present data on risk of bias of each study and, if available, any outcome level assessment.	***Supplementary Materials page 10***
Results of individual studies	20	For all outcomes considered (benefits or harms), present, for each study: 1) simple summary data for each	***5~8***

		intervention group, and 2) effect estimates and confidence intervals. *Modified approaches may be needed to deal with information from larger networks.*	
Synthesis of results	21	Present results of each meta-analysis done, including confidence/credible intervals. *In larger networks, authors may focus on comparisons versus a particular comparator (e.g. placebo or standard care), with full findings presented in an appendix. League tables and forest plots may be considered to summarize pairwise comparisons.* If additional summary measures were explored (such as treatment rankings), these should also be presented.	***6~9***
**Exploration for inconsistency**	**S5**	Describe results from investigations of inconsistency. This may include such information as measures of model fit to compare consistency and inconsistency models, *P* values from statistical tests, or summary of inconsistency estimates from different parts of the treatment network.	/
Risk of bias across studies	22	Present results of any assessment of risk of bias across studies for the evidence base being studied.	***Supplementary Materials page 10***
Results of additional analyses	23	Give results of additional analyses, if done (e.g., sensitivity or subgroup analyses, meta-regression analyses, *alternative network geometries studied, alternative choice of prior distributions for Bayesian analyses,* and so forth).	***8~9***
**DISCUSSION**			
Summary of evidence	24	Summarize the main findings, including the strength of evidence for each main outcome; consider their relevance to key groups (e.g., healthcare providers, users, and policy-makers).	***9~10***

Limitations	25	Discuss limitations at study and outcome level (e.g., risk of bias), and at review level (e.g., incomplete retrieval of identified research, reporting bias). *Comment on the validity of the assumptions, such as transitivity and consistency. Comment on any concerns regarding network geometry (e.g., avoidance of certain comparisons).*	***10***
Conclusions	26	Provide a general interpretation of the results in the context of other evidence, and implications for future research.	***11***
**FUNDING**			
Funding	27	Describe sources of funding for the systematic review and other support (e.g., supply of data); role of funders for the systematic review. This should also include information regarding whether funding has been received from manufacturers of treatments in the network and/or whether some of the authors are content experts with professional conflicts of interest that could affect use of treatments in the network.	***11***

PRISMA = Preferred Reporting Items for Systematic Reviews and Meta-Analysis; PICOS = population, intervention, comparators, outcomes, study design.

* Text in italics indicates wording specific to reporting of network meta-analyses that has been added to guidance from the PRISMA statement.

† Authors may wish to plan for use of appendices to present all relevant information in full detail for items in this section.

Table S2: Retrieval strategy for the network meta-analysis in MEDLINE

#	Retrieval strategy	Results
1	"randomized controlled trial".pt.	571765
2	(random$ or placebo$ or single blind$ or double blind$ or triple blind$).ti,ab.	1235396
3	(retraction of publication or retracted publication).pt.	8429
4	or/1-3	1346617
5	(animals not humans).sh.	4990035
6	((comment or editorial or meta-analysis or practice-guideline or review or letter or journal correspondence) not "randomized controlled trial").pt.	4737560
7	(random sampl$ or random digit$ or random effect$ or random survey or random regression).ti,ab. not "randomized controlled trial".pt.	97041
8	4 not (5 or 6 or 7)	950021
9	exp Carcinoma, Non-Small-Cell Lung/ or ('non-small-cell lung cancer' or 'non-small cell lung cancer' or 'non small-cell lung cancer' or 'non small cell lung cancer' or 'non-small-cell lung carcinoma' or 'non-small cell lung carcinoma' or 'non small-cell lung carcinoma' or 'non small cell lung carcinoma' or nsclc).ti,ab.	75062
10	exp ErbB Receptors/ or ('ErbB Receptors' or 'Epidermal Growth Factor Receptor' or 'EGF Receptors' or 'EGFR').ti,ab.	107428
11	exp "Molecular Targeted Therapies"/ or exp 'tyrosine kinase inhibitor'/ or exp 'TKI'/ or ('gefitinib' or 'Iressa' or 'icotinib' or 'erlotinib' or 'Erlotinib Hydrochloride' or 'Tarceva' or 'afatinib' or 'Gilotrif' or 'dacomitinib' or 'Vizimpro' or 'osimertinib' or 'Tagrisso').ti,ab.	45790
12	exp Drug Therapy, Combination/ or (combin* or plus or add* or alone).ti,ab.	5471392
13	8 and 9 and 10 and 11 and 12	328

Unique ID	Study ID	Experimental	Comparator	Outcome	Weight	D1	D2	D3	D4	D5	Overall
Y. Cheng 2016	NCT01469000	Gefitinib−Pemetrexed	Gefitinib	PFS	1	+	!	+	!	!	!
B. Han 2017	NCT02148380	Gefitinib−Pemetrexed&C	Gefitinib	PFS	1	!	!	+	!	+	!
L. Xu 2019	NCT02031601	Icotinib+Pemetrexed&Ca	Icotinib	PFS	1	+	+	+	+	!	!
Y. Hosomi 2019	UMIN000006340	Gefitinib−Pemetrexed&C	Gefitinib	PFS	1	+	+	+	!	+	!
V. Noronha 2019	CTRI/2016/08/007149	Gefitinib−Pemetrexed&C	Gefitinib	PFS	1	+	+	+	!	+	!
T. Seto 2014	JapicCTI-111390	Erlotinib+Bevacizumab	Erlotinib	PFS	1	+	!	+	+	+	!
H. Saito 2019	UMIN000017069	Erlotinib+Bevacizumab	Erlotinib	PFS	1	+	!	+	+	+	!
T. E. Stinchcombe 201	NCT01532089	Erlotinib+Bevacizumab	Erlotinib	PFS	1	!	!	+	!	+	!
Q. Zhou 2021	NCT02759614	Erlotinib+Bevacizumab	Erlotinib	PFS	1	!	!	+	+	!	!
M. C. Piccirillo 2022	NCT02633189	Erlotinib+Bevacizumab	Erlotinib	PFS	1	!	!	+	+	!	!
K. Nakagawa 2019	NCT02411448	Erlotinib+Ramucirumab	Erlotinib−Placebo	PFS	1	+	+	+	+	+	+
H. Zhao 2021	NCT02824458	Gefitinib−Apatinib	Gefitinib+Placebo	PFS	1	!	+	+	+	+	!
N. B. Leighl 2017	NCT01221077	Erlotinib+Linsitinib	Erlotinib−Placebo	PFS	1	+	+	+	+	+	+
L. Li 2019	NCT03071705	Gefitinib−Metformin	Gefitinib+Placebo	PFS	1	+	+	!	+	+	!
R. G. Campelo 2020	NCT01513174	Gefitinib Olaparib	Gefitinib	PFS	1	!	!	+	!	+	!
X. Wang 2022	NCT02893332	Gefitinib/Erlotinib/Icotinib	Gefitinib/Erlotinib/Icotinib	PFS	1	!	!	+	!	+	!
S. B. Goldberg 2020	NCT02438722	Gefitinib−Cetuximab	Gefitinib	PFS	1	+	!	+	!	+	!
H. Kenmotsu 2022	UMIN000030206	Osimertinib+Bevacizumal	Osimertinib	PFS	1	!	!	+	+	+	!
C. An 2016	null	Gefitinib−Pemetrexed	Gefitinib+Placebo	ORR	1	+	+	+	+	!	!
X. Gu 2011	null	Gefitinib−Cryoablation	Gefitinib	ORR	1	!	+	+	!	!	!
B. Yu 2019	null	Gefitinib Microwave abl	Gefitinib	ORR	1	!	+	+	!	!	!
X. Zheng 2016	null	Erlotinib/Icotinib Radiatio	Erlotinib/Icotinib	ORR	1	!	+	+	!	!	!
Y. Qiu 2020	null	Icotinib+Radiotherapy&C	Icotinib	ORR	1	!	+	+	!	!	!
A. B. Cortot 2021	NCT02716311	Afatinib+Cetuximab	Afatinib	ORR	1	+	!	+	!	+	!

+ Low risk
! Some concerns
− High risk

D1 Randomisation process
D2 Deviations from the intended interventions
D3 Missing outcome data
D4 Measurement of the outcome
D5 Selection of the reported result

Figure S1. Assessment of risk of bias by version 2 of the Cochrane tool for assessing the risk of bias in randomized trial (RoB2)

A

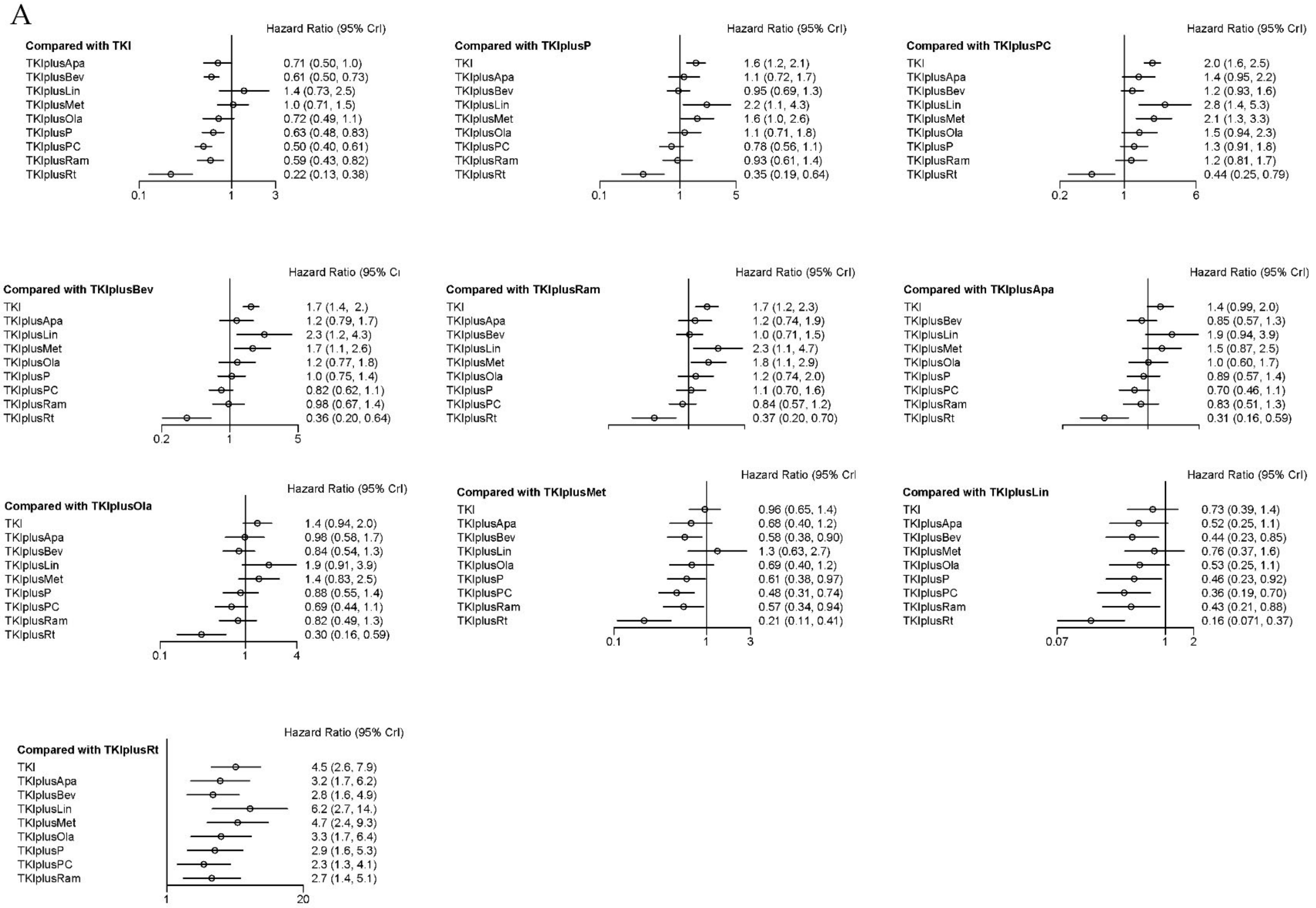
Compared with TKI
Hazard Ratio (95% CrI)
TKIplusApa 0.71 (0.50, 1.0)
TKIplusBev 0.61 (0.50, 0.73)
TKIplusLin 1.4 (0.73, 2.5)
TKIplusMet 1.0 (0.71, 1.5)
TKIplusOla 0.72 (0.49, 1.1)
TKIplusP 0.63 (0.48, 0.83)
TKIplusPC 0.50 (0.40, 0.61)
TKIplusRam 0.59 (0.43, 0.82)
TKIplusRt 0.22 (0.13, 0.38)
0.1
1
3
Compared with TKIplusP
Hazard Ratio (95% CrI)
TKI 1.6 (1.2, 2.1)
TKIplusApa 1.1 (0.72, 1.7)
TKIplusBev 0.95 (0.69, 1.3)
TKIplusLin 2.2 (1.1, 4.3)
TKIplusMet 1.6 (1.0, 2.6)
TKIplusOla 1.1 (0.71, 1.8)
TKIplusPC 0.78 (0.56, 1.1)
TKIplusRam 0.93 (0.61, 1.4)
TKIplusRt 0.35 (0.19, 0.64)
0.1
1
5
Compared with TKIplusPC
Hazard Ratio (95% CrI)
TKI 2.0 (1.6, 2.5)
TKIplusApa 1.4 (0.95, 2.2)
TKIplusBev 1.2 (0.93, 1.6)
TKIplusLin 2.8 (1.4, 5.3)
TKIplusMet 2.1 (1.3, 3.3)
TKIplusOla 1.5 (0.94, 2.3)
TKIplusP 1.3 (0.91, 1.8)
TKIplusRam 1.2 (0.81, 1.7)
TKIplusRt 0.44 (0.25, 0.79)
0.2
1
6
Compared with TKIplusBev
Hazard Ratio (95% Cı
TKI 1.7 (1.4, 2.)
TKIplusApa 1.2 (0.79, 1.7)
TKIplusLin 2.3 (1.2, 4.3)
TKIplusMet 1.7 (1.1, 2.6)
TKIplusOla 1.2 (0.77, 1.8)
TKIplusP 1.0 (0.75, 1.4)
TKIplusPC 0.82 (0.62, 1.1)
TKIplusRam 0.98 (0.67, 1.4)
TKIplusRt 0.36 (0.20, 0.64)
0.2
1
5
Compared with TKIplusRam
Hazard Ratio (95% CrI)
TKI 1.7 (1.2, 2.3)
TKIplusApa 1.2 (0.74, 1.9)
TKIplusBev 1.0 (0.71, 1.5)
TKIplusLin 2.3 (1.1, 4.7)
TKIplusMet 1.8 (1.1, 2.9)
TKIplusOla 1.2 (0.74, 2.0)
TKIplusP 1.1 (0.70, 1.6)
TKIplusPC 0.84 (0.57, 1.2)
TKIplusRt 0.37 (0.20, 0.70)
Compared with TKIplusApa
Hazard Ratio (95% CrI)
TKI 1.4 (0.99, 2.0)
TKIplusBev 0.85 (0.57, 1.3)
TKIplusLin 1.9 (0.94, 3.9)
TKIplusMet 1.5 (0.87, 2.5)
TKIplusOla 1.0 (0.60, 1.7)
TKIplusP 0.89 (0.57, 1.4)
TKIplusPC 0.70 (0.46, 1.1)
TKIplusRam 0.83 (0.51, 1.3)
TKIplusRt 0.31 (0.16, 0.59)
Compared with TKIplusOla
Hazard Ratio (95% CrI)
TKI 1.4 (0.94, 2.0)
TKIplusApa 0.98 (0.58, 1.7)
TKIplusBev 0.84 (0.54, 1.3)
TKIplusLin 1.9 (0.91, 3.9)
TKIplusMet 1.4 (0.83, 2.5)
TKIplusP 0.88 (0.55, 1.4)
TKIplusPC 0.69 (0.44, 1.1)
TKIplusRam 0.82 (0.49, 1.3)
TKIplusRt 0.30 (0.16, 0.59)
0.1
1
4
Compared with TKIplusMet
Hazard Ratio (95% CrI
TKI 0.96 (0.65, 1.4)
TKIplusApa 0.68 (0.40, 1.2)
TKIplusBev 0.58 (0.38, 0.90)
TKIplusLin 1.3 (0.63, 2.7)
TKIplusOla 0.69 (0.40, 1.2)
TKIplusP 0.61 (0.38, 0.97)
TKIplusPC 0.48 (0.31, 0.74)
TKIplusRam 0.57 (0.34, 0.94)
TKIplusRt 0.21 (0.11, 0.41)
0.1
1
3
Compared with TKIplusLin
Hazard Ratio (95% CrI)
TKI 0.73 (0.39, 1.4)
TKIplusApa 0.52 (0.25, 1.1)
TKIplusBev 0.44 (0.23, 0.85)
TKIplusMet 0.76 (0.37, 1.6)
TKIplusOla 0.53 (0.25, 1.1)
TKIplusP 0.46 (0.23, 0.92)
TKIplusPC 0.36 (0.19, 0.70)
TKIplusRam 0.43 (0.21, 0.88)
TKIplusRt 0.16 (0.071, 0.37)
0.07
1
2
Compared with TKIplusRt
Hazard Ratio (95% CrI)
TKI 4.5 (2.6, 7.9)
TKIplusApa 3.2 (1.7, 6.2)
TKIplusBev 2.8 (1.6, 4.9)
TKIplusLin 6.2 (2.7, 14.)
TKIplusMet 4.7 (2.4, 9.3)
TKIplusOla 3.3 (1.7, 6.4)
TKIplusP 2.9 (1.6, 5.3)
TKIplusPC 2.3 (1.3, 4.1)
TKIplusRam 2.7 (1.4, 5.1)
1
20

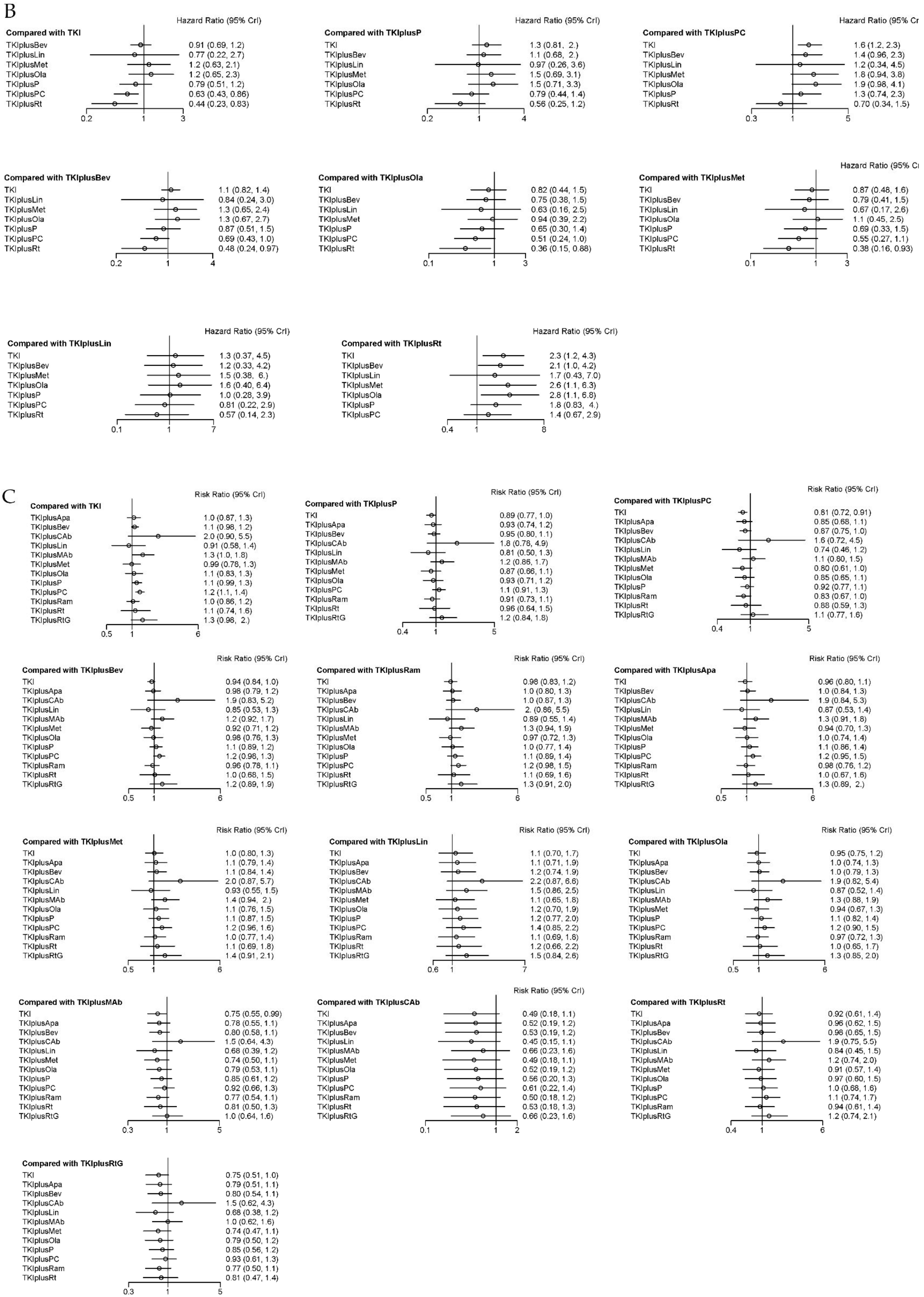
B
Compared with TKI
Hazard Ratio (95% CrI)
TKIplusBev 0.91 (0.69, 1.2)
TKIplusLin 0.77 (0.22, 2.7)
TKIplusMet 1.2 (0.63, 2.1)
TKIplusOla 1.2 (0.65, 2.3)
TKIplusP 0.79 (0.51, 1.2)
TKIplusPC 0.63 (0.43, 0.86)
TKIplusRt 0.44 (0.23, 0.83)
0.2 1 3
Compared with TKIplusP
Hazard Ratio (95% CrI)
TKI 1.3 (0.81, 2.)
TKIplusBev 1.1 (0.68, 2.)
TKIplusLin 0.97 (0.26, 3.6)
TKIplusMet 1.5 (0.69, 3.1)
TKIplusOla 1.5 (0.71, 3.3)
TKIplusPC 0.79 (0.44, 1.4)
TKIplusRt 0.56 (0.25, 1.2)
0.2 1 4
Compared with TKIplusPC
Hazard Ratio (95% Cr
TKI 1.6 (1.2, 2.3)
TKIplusBev 1.4 (0.96, 2.3)
TKIplusLin 1.2 (0.34, 4.5)
TKIplusMet 1.8 (0.94, 3.8)
TKIplusOla 1.9 (0.98, 4.1)
TKIplusP 1.3 (0.74, 2.3)
TKIplusRt 0.70 (0.34, 1.5)
0.3 1 5
Compared with TKIplusBev
TKI 1.1 (0.82, 1.4)
TKIplusLin 0.84 (0.24, 3.0)
TKIplusMet 1.3 (0.65, 2.4)
TKIplusOla 1.3 (0.67, 2.7)
TKIplusP 0.87 (0.51, 1.5)
TKIplusPC 0.69 (0.43, 1.0)
TKIplusRt 0.48 (0.24, 0.97)
0.2 1 4
Compared with TKIplusOla
TKI 0.82 (0.44, 1.5)
TKIplusBev 0.75 (0.38, 1.5)
TKIplusLin 0.63 (0.16, 2.5)
TKIplusMet 0.94 (0.39, 2.2)
TKIplusP 0.65 (0.30, 1.4)
TKIplusPC 0.51 (0.24, 1.0)
TKIplusRt 0.36 (0.15, 0.88)
0.1 1 3
Compared with TKIplusMet
Hazard Ratio (95% Cr
TKI 0.87 (0.48, 1.6)
TKIplusBev 0.79 (0.41, 1.5)
TKIplusLin 0.67 (0.17, 2.6)
TKIplusOla 1.1 (0.45, 2.5)
TKIplusP 0.69 (0.33, 1.5)
TKIplusPC 0.55 (0.27, 1.1)
TKIplusRt 0.38 (0.16, 0.93)
0.1 1 3
Compared with TKIplusLin
Hazard Ratio (95% CrI)
TKI 1.3 (0.37, 4.5)
TKIplusBev 1.2 (0.33, 4.2)
TKIplusMet 1.5 (0.38, 6.)
TKIplusOla 1.6 (0.40, 6.4)
TKIplusP 1.0 (0.28, 3.9)
TKIplusPC 0.81 (0.22, 2.9)
TKIplusRt 0.57 (0.14, 2.3)
0.1 1 7
Compared with TKIplusRt
Hazard Ratio (95% CrI)
TKI 2.3 (1.2, 4.3)
TKIplusBev 2.1 (1.0, 4.2)
TKIplusLin 1.7 (0.43, 7.0)
TKIplusMet 2.6 (1.1, 6.3)
TKIplusOla 2.8 (1.1, 6.8)
TKIplusP 1.8 (0.83, 4.)
TKIplusPC 1.4 (0.67, 2.9)
0.4 1 8
C
Compared with TKI
Risk Ratio (95% CrI)
TKIplusApa 1.0 (0.87, 1.3)
TKIplusBev 1.1 (0.98, 1.2)
TKIplusCAb 2.0 (0.90, 5.5)
TKIplusLin 0.91 (0.58, 1.4)
TKIplusMAb 1.3 (1.0, 1.8)
TKIplusMet 0.99 (0.78, 1.3)
TKIplusOla 1.1 (0.83, 1.3)
TKIplusP 1.1 (0.99, 1.3)
TKIplusPC 1.2 (1.1, 1.4)
TKIplusRam 1.0 (0.86, 1.2)
TKIplusRt 1.1 (0.74, 1.6)
TKIplusRtG 1.3 (0.96, 2.)
0.5 1 6
Compared with TKIplusP
Risk Ratio (95% CrI)
TKI 0.89 (0.77, 1.0)
TKIplusApa 0.93 (0.74, 1.2)
TKIplusBev 0.95 (0.80, 1.1)
TKIplusCAb 1.8 (0.78, 4.9)
TKIplusLin 0.81 (0.50, 1.3)
TKIplusMAb 1.2 (0.86, 1.7)
TKIplusMet 0.87 (0.66, 1.1)
TKIplusOla 0.93 (0.71, 1.2)
TKIplusPC 1.1 (0.91, 1.3)
TKIplusRam 0.91 (0.73, 1.1)
TKIplusRt 0.96 (0.64, 1.5)
TKIplusRtG 1.2 (0.84, 1.8)
0.4 1 5
Compared with TKIplusPC
Risk Ratio (95% CrI)
TKI 0.81 (0.72, 0.91)
TKIplusApa 0.85 (0.68, 1.1)
TKIplusBev 0.87 (0.75, 1.0)
TKIplusCAb 1.6 (0.72, 4.5)
TKIplusLin 0.74 (0.46, 1.2)
TKIplusMAb 1.1 (0.80, 1.5)
TKIplusMet 0.80 (0.61, 1.0)
TKIplusOla 0.85 (0.65, 1.1)
TKIplusP 0.92 (0.77, 1.1)
TKIplusRam 0.83 (0.67, 1.0)
TKIplusRt 0.88 (0.59, 1.3)
TKIplusRtG 1.1 (0.77, 1.6)
0.4 1 5
Compared with TKIplusBev
Risk Ratio (95% CrI)
TKI 0.94 (0.84, 1.0)
TKIplusApa 0.98 (0.79, 1.2)
TKIplusCAb 1.9 (0.83, 5.2)
TKIplusLin 0.85 (0.53, 1.3)
TKIplusMAb 1.2 (0.92, 1.7)
TKIplusMet 0.92 (0.71, 1.2)
TKIplusOla 0.98 (0.76, 1.3)
TKIplusP 1.1 (0.89, 1.2)
TKIplusPC 1.2 (0.98, 1.3)
TKIplusRam 0.96 (0.78, 1.1)
TKIplusRt 1.0 (0.68, 1.5)
TKIplusRtG 1.2 (0.89, 1.9)
0.5 1 6
Compared with TKIplusRam
Risk Ratio (95% CrI)
TKI 0.98 (0.83, 1.2)
TKIplusApa 1.0 (0.80, 1.3)
TKIplusBev 1.0 (0.87, 1.3)
TKIplusCAb 2. (0.86, 5.5)
TKIplusLin 0.89 (0.55, 1.4)
TKIplusMAb 1.3 (0.94, 1.9)
TKIplusMet 0.97 (0.72, 1.3)
TKIplusOla 1.0 (0.77, 1.4)
TKIplusP 1.1 (0.89, 1.4)
TKIplusPC 1.2 (0.98, 1.5)
TKIplusRt 1.1 (0.69, 1.6)
TKIplusRtG 1.3 (0.91, 2.0)
0.5 1 6
Compared with TKIplusApa
Risk Ratio (95% CrI)
TKI 0.96 (0.80, 1.1)
TKIplusBev 1.0 (0.84, 1.3)
TKIplusCAb 1.9 (0.84, 5.3)
TKIplusLin 0.87 (0.53, 1.4)
TKIplusMAb 1.3 (0.91, 1.8)
TKIplusMet 0.94 (0.70, 1.3)
TKIplusOla 1.0 (0.74, 1.4)
TKIplusP 1.1 (0.86, 1.4)
TKIplusPC 1.2 (0.95, 1.5)
TKIplusRam 0.98 (0.76, 1.2)
TKIplusRt 1.0 (0.67, 1.6)
TKIplusRtG 1.3 (0.89, 2.)
0.5 1 6
Compared with TKIplusMet
Risk Ratio (95% CrI)
TKI 1.0 (0.80, 1.3)
TKIplusApa 1.1 (0.79, 1.4)
TKIplusBev 1.1 (0.84, 1.4)
TKIplusCAb 2.0 (0.87, 5.7)
TKIplusLin 0.93 (0.55, 1.5)
TKIplusMAb 1.4 (0.94, 2.)
TKIplusOla 1.1 (0.76, 1.5)
TKIplusP 1.1 (0.87, 1.5)
TKIplusPC 1.2 (0.96, 1.6)
TKIplusRam 1.0 (0.77, 1.4)
TKIplusRt 1.1 (0.69, 1.8)
TKIplusRtG 1.4 (0.91, 2.1)
0.5 1 6
Compared with TKIplusLin
Risk Ratio (95% CrI)
TKI 1.1 (0.70, 1.7)
TKIplusApa 1.1 (0.71, 1.9)
TKIplusBev 1.2 (0.74, 1.9)
TKIplusCAb 2.2 (0.87, 6.6)
TKIplusMAb 1.5 (0.86, 2.5)
TKIplusMet 1.1 (0.65, 1.8)
TKIplusOla 1.2 (0.70, 1.9)
TKIplusP 1.2 (0.77, 2.0)
TKIplusPC 1.4 (0.85, 2.2)
TKIplusRam 1.1 (0.69, 1.8)
TKIplusRt 1.2 (0.66, 2.2)
TKIplusRtG 1.5 (0.84, 2.6)
0.6 1 7
Compared with TKIplusOla
Risk Ratio (95% CrI)
TKI 0.95 (0.75, 1.2)
TKIplusApa 1.0 (0.74, 1.3)
TKIplusBev 1.0 (0.79, 1.3)
TKIplusCAb 1.9 (0.82, 5.4)
TKIplusLin 0.87 (0.52, 1.4)
TKIplusMAb 1.3 (0.88, 1.9)
TKIplusMet 0.94 (0.67, 1.3)
TKIplusP 1.1 (0.82, 1.4)
TKIplusPC 1.2 (0.90, 1.5)
TKIplusRam 0.97 (0.72, 1.3)
TKIplusRt 1.0 (0.65, 1.7)
TKIplusRtG 1.3 (0.85, 2.0)
0.5 1 6
Compared with TKIplusMAb
TKI 0.75 (0.55, 0.99)
TKIplusApa 0.78 (0.55, 1.1)
TKIplusBev 0.80 (0.58, 1.1)
TKIplusCAb 1.5 (0.64, 4.3)
TKIplusLin 0.68 (0.39, 1.2)
TKIplusMet 0.74 (0.50, 1.1)
TKIplusOla 0.79 (0.53, 1.1)
TKIplusP 0.85 (0.61, 1.2)
TKIplusPC 0.92 (0.66, 1.3)
TKIplusRam 0.77 (0.54, 1.1)
TKIplusRt 0.81 (0.50, 1.3)
TKIplusRtG 1.0 (0.64, 1.6)
0.3 1 5
Compared with TKIplusCAb
Risk Ratio (95% CrI)
TKI 0.49 (0.18, 1.1)
TKIplusApa 0.52 (0.19, 1.2)
TKIplusBev 0.53 (0.19, 1.2)
TKIplusLin 0.45 (0.15, 1.1)
TKIplusMAb 0.66 (0.23, 1.6)
TKIplusMet 0.49 (0.18, 1.1)
TKIplusOla 0.52 (0.19, 1.2)
TKIplusP 0.56 (0.20, 1.3)
TKIplusPC 0.61 (0.22, 1.4)
TKIplusRam 0.50 (0.18, 1.2)
TKIplusRt 0.53 (0.18, 1.3)
TKIplusRtG 0.66 (0.23, 1.6)
0.1 1 2
Compared with TKIplusRt
TKI 0.92 (0.61, 1.4)
TKIplusApa 0.96 (0.62, 1.5)
TKIplusBev 0.96 (0.65, 1.5)
TKIplusCAb 1.9 (0.75, 5.5)
TKIplusLin 0.84 (0.45, 1.5)
TKIplusMAb 1.2 (0.74, 2.0)
TKIplusMet 0.91 (0.57, 1.4)
TKIplusOla 0.97 (0.60, 1.5)
TKIplusP 1.0 (0.68, 1.6)
TKIplusPC 1.1 (0.74, 1.7)
TKIplusRam 0.94 (0.61, 1.4)
TKIplusRtG 1.2 (0.74, 2.1)
0.4 1 6
Compared with TKIplusRtG
TKI 0.75 (0.51, 1.0)
TKIplusApa 0.79 (0.51, 1.1)
TKIplusBev 0.80 (0.54, 1.1)
TKIplusCAb 1.5 (0.62, 4.3)
TKIplusLin 0.68 (0.38, 1.2)
TKIplusMAb 1.0 (0.62, 1.6)
TKIplusMet 0.74 (0.47, 1.1)
TKIplusOla 0.79 (0.50, 1.2)
TKIplusP 0.85 (0.56, 1.2)
TKIplusPC 0.93 (0.61, 1.3)
TKIplusRam 0.77 (0.50, 1.1)
TKIplusRt 0.81 (0.47, 1.4)
0.3 1 5

D

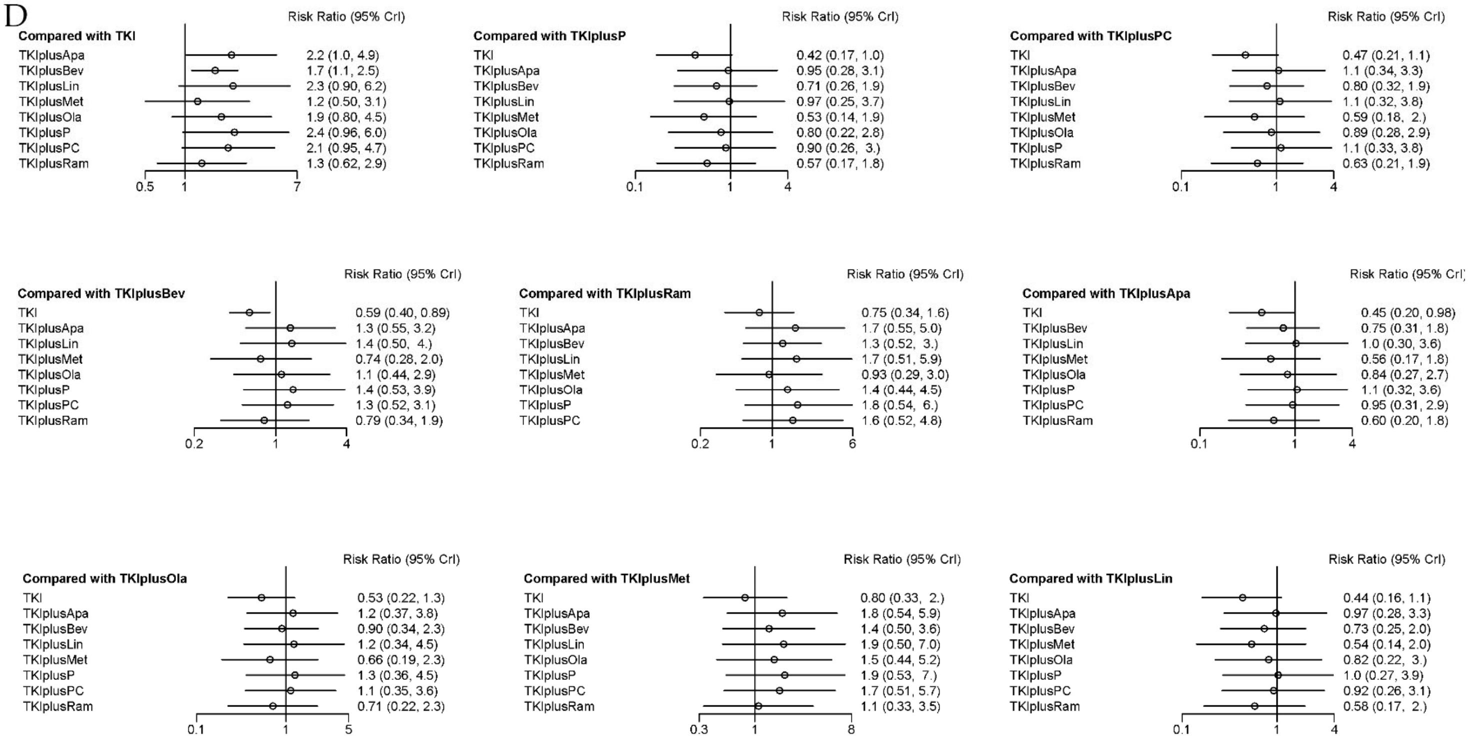

Figure S2. Forest plots of one treatment's comparison with other treatments

(A) Progression free survival (PFS) of one treatment was compared with other enrolled treatments. (B) Overall survival (OS) of one treatment was compared with other enrolled treatments. (C) Objective response rate (ORR) of one treatment was compared with other enrolled treatments. (D) Adverse events of grade 3 or higher (≥Grade 3 AEs) of one treatment was compared with other enrolled treatments.

TKI: tyrosine kinase inhibitors, representing first-generation EGFR-TKIs in this network meta-analysis (including gefitinib, erlotinib, and icotinib). TKIplusP: TKI plus pemetrexed; TKIplusPC: TKI plus pemetrexed & carboplatin; TKIplusBev: TKI plus bevacizumab; TKIplusRam: TKI plus ramucirumab; TKIplusApa: TKI plus apatinib; TKIplusCAb: TKI plus cryoablation; TKIplusMAb: TKI plus microwave ablation; TKIplusLin: TKI plus linsitinib; TKIplusMet: TKI plus metformin; TKIplusOla: TKI plus olaparib; TKIplusRt: TKI plus radiation; TKIplusRtG: TKI plus radiotherapy & GM-CSF

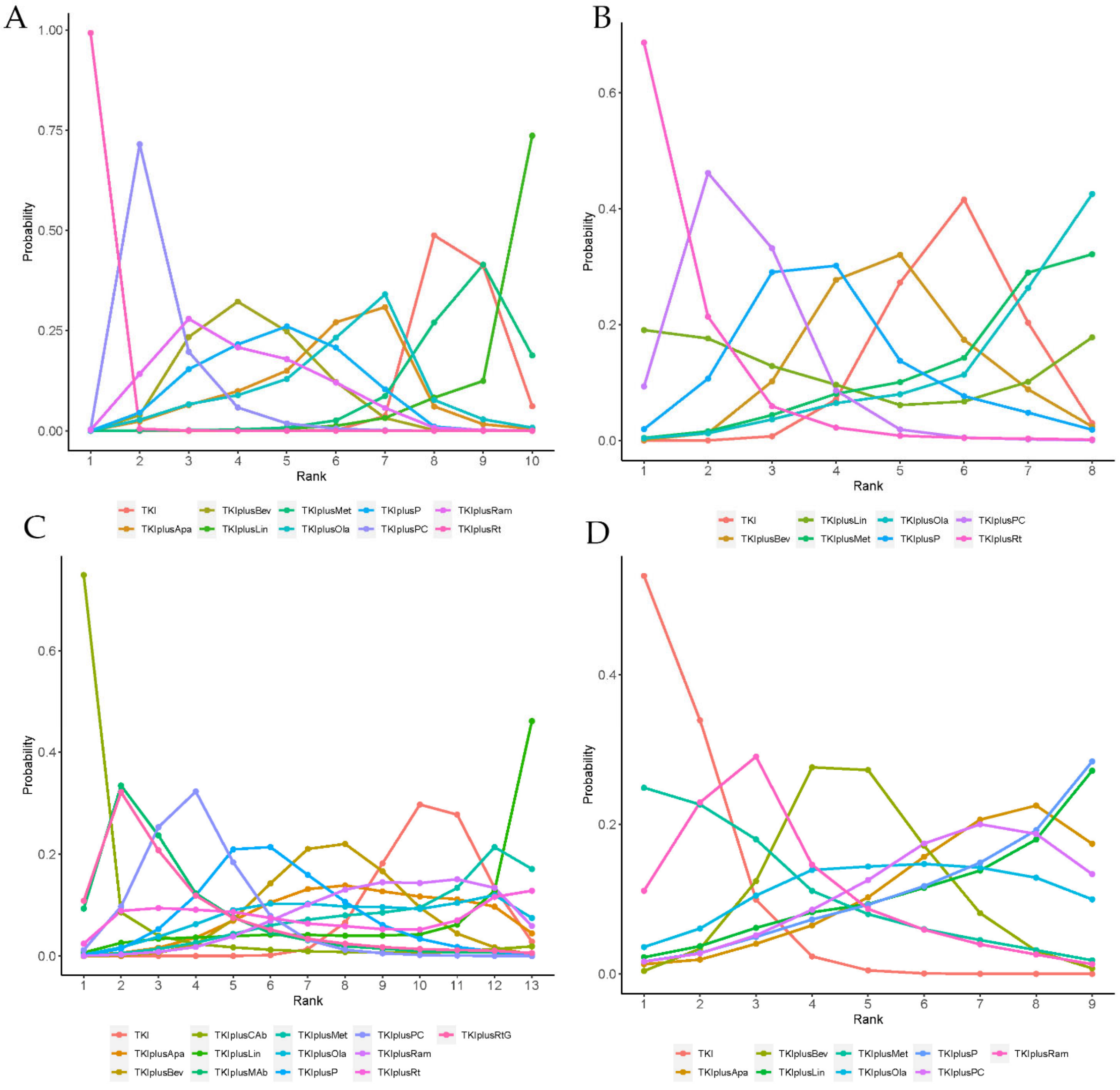

Figure S3. Diagrams showing the probability of ranks for each treatment.

The different color represents different treatment. The abscissa represents the ranking and the ordinate represents the probability that the treatment can rank the position corresponding to its abscissa. (A) Progression free survival (PFS). (B) Overall survival (OS). (C) Objective response rate (ORR). (D) Adverse events of grade 3 or higher (≥Grade 3 AEs). TKI=Tyrosine kinase inhibitors (TKI) monotherapy; TKIplusP=TKI plus pemetrexed; TKIplusPC=TKI plus pemetrexed & carboplatin; TKIplusBev=TKI plus bevacizumab; TKIplusRam=TKI plus ramucirumab; TKIplusApa=TKI plus apatinib; TKIplusCAb=TKI plus cryoablation; TKIplusMAb=TKI plus microwave ablation; TKIplusLin=TKI plus linsitinib; TKIplusMet=TKI plus metformin; TKIplusOla=TKI plus olaparib; TKIplusRt=TKI plus radiation; TKIplusRtG=TKI plus radiotherapy & GM-CSF.

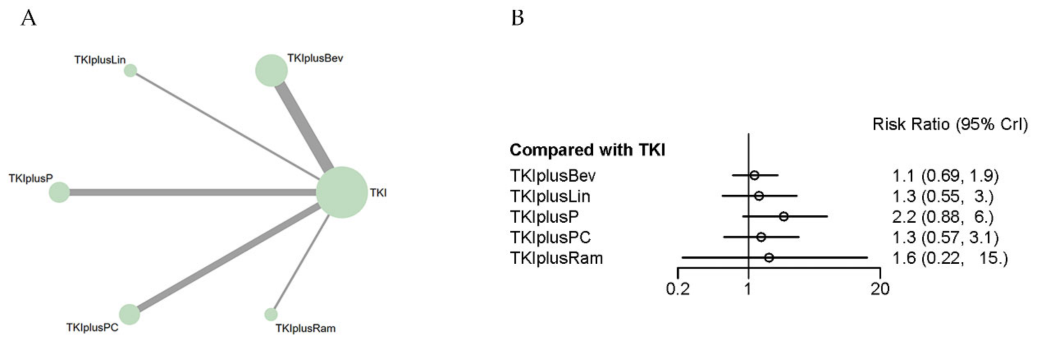

Figure S4. The network plot and forest plot of interruption rate of TKI owing to AEs of different treatments.

(A) The network plot; (B) forest plot.

TKI=Tyrosine kinase inhibitors (TKI) monotherapy; TKIplusP=TKI plus pemetrexed; TKIplusPC=TKI plus pemetrexed & carboplatin; TKIplusBev=TKI plus bevacizumab; TKIplusRam=TKI plus ramucirumab; TKIplusLin=TKI plus linsitinib.

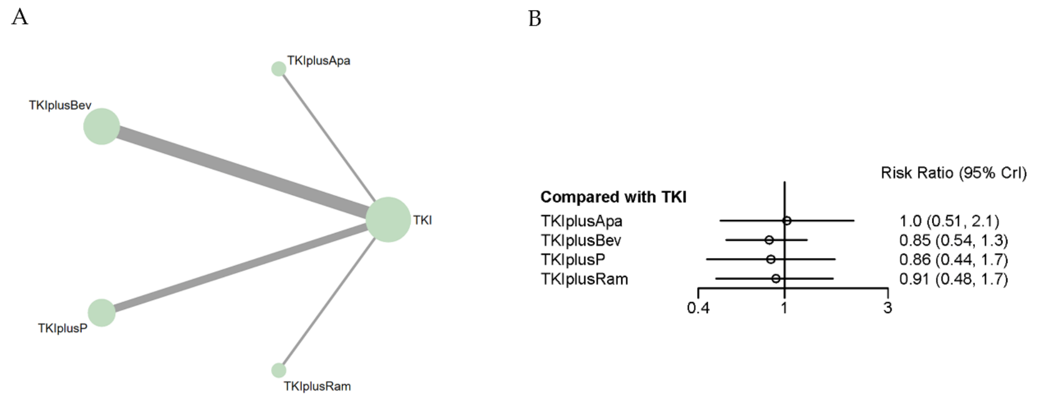

Figure S5. The network plot and forest plot of the incidence of developing T790M mutation in patients with the first generation TKIs resistance of different treatments.

(A)The network plot; (B) forest plot.

TKI=Tyrosine kinase inhibitors (TKI) monotherapy; TKIplusP=TKI plus pemetrexed; TKIplusBev=TKI plus bevacizumab; TKIplusRam=TKI plus ramucirumab; TKIplusApa=TKI plus apatinib.

Figure S6. Network plots and ranking diagrams in subgroups.

A

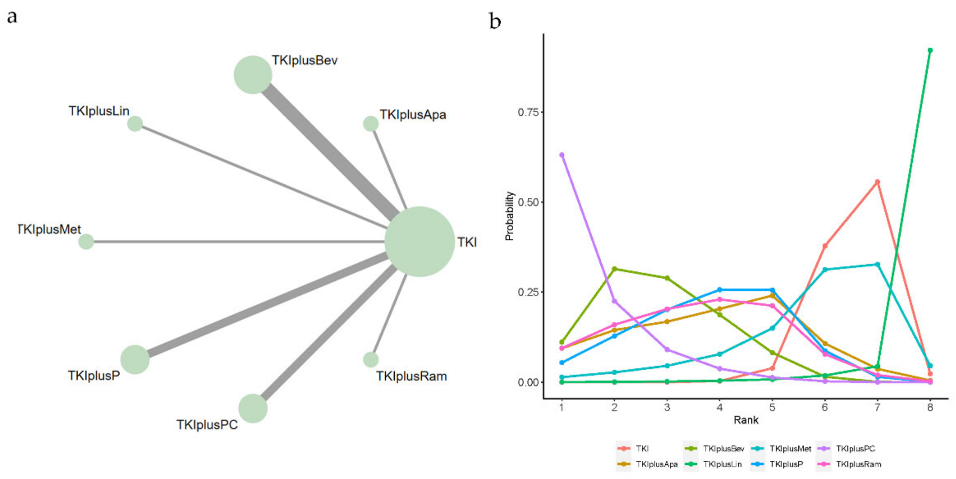

A. (a). Network diagram of comparisons on progression-free survival in patients with EGFR 19deltion mutation; (b). Rank profile of comparisons on progression-free survival in patients with EGFR 19deltion mutation.

B

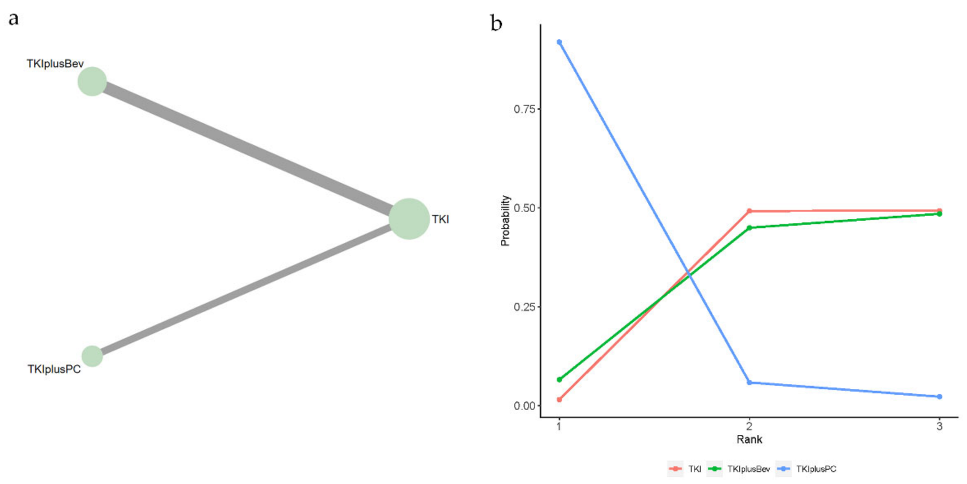

B. (a). Network diagram of comparisons on overall survival in patients with EGFR 19deltion mutation; (b). Rank profile of comparisons on overall survival in patients with EGFR 19deltion mutation.

C

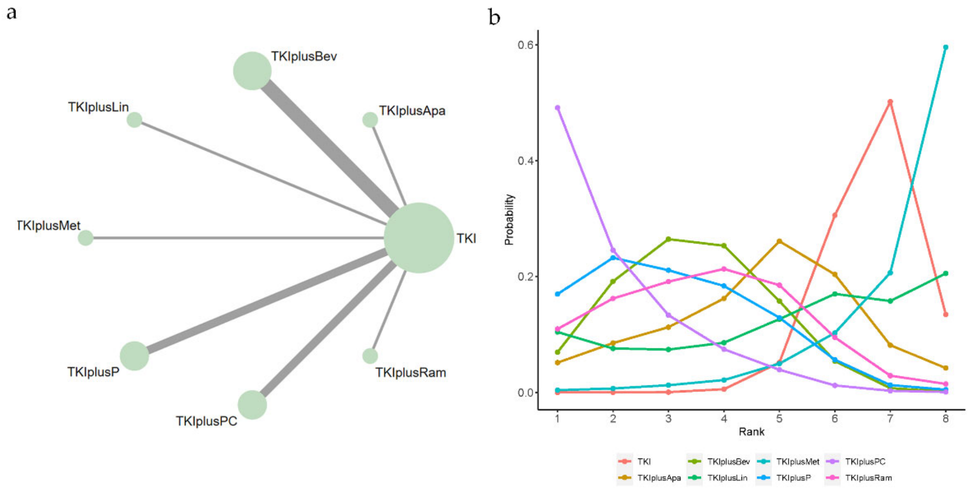

C. (a). Network diagram of comparisons on progression-free survival in patients with EGFR Leu858Arg mutation; (b). Rank profile of comparisons on progression-free survival in patients with EGFR Leu858Arg mutation.

D

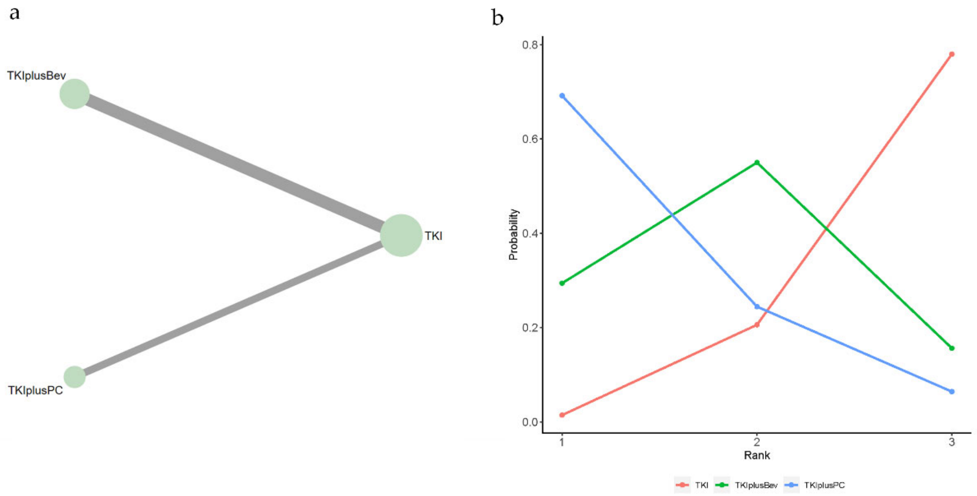

D. (a). Network diagram of comparisons on overall survival in patients with EGFR Leu858Arg mutation; (b). Rank profile of comparisons on overall survival in patients with EGFR Leu858Arg mutation.

E

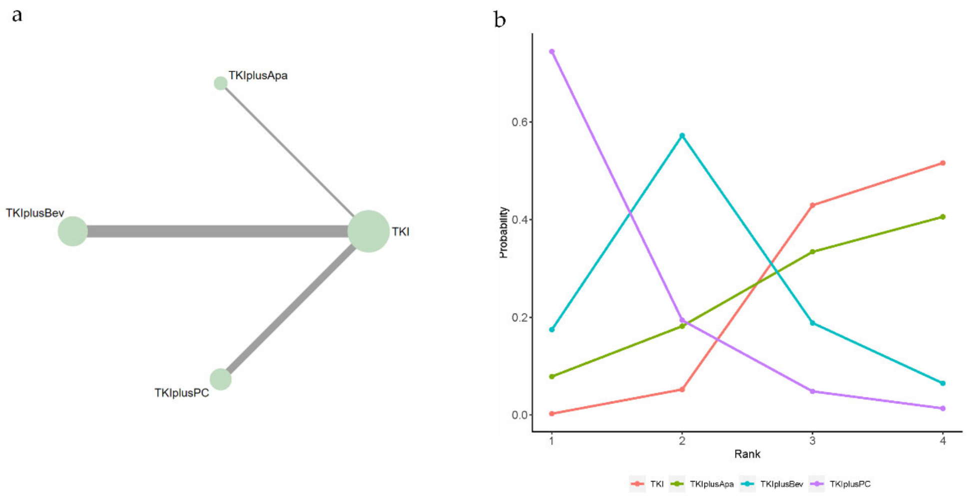

E. (a). Network diagram of comparisons on progression-free survival in patients with brain metastasis; (b). Rank profile of comparisons on progression-free survival in patients with brain metastasis.

F

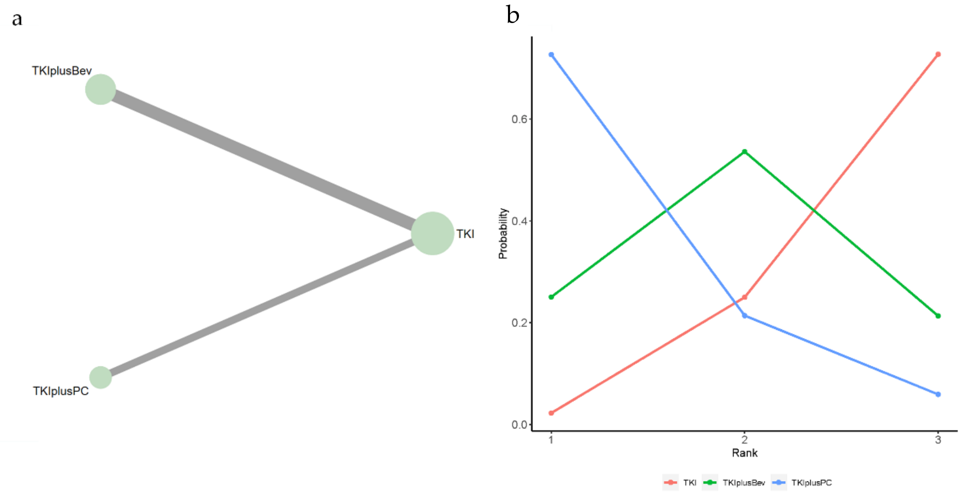

F. (a). Network diagram of comparisons on overall survival in patients with brain metastasis; (b). Rank profile of comparisons on overall survival in patients with brain metastasis.

G

a

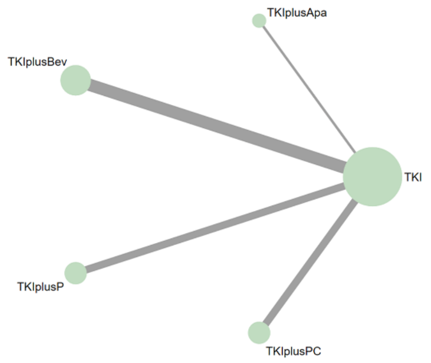

b

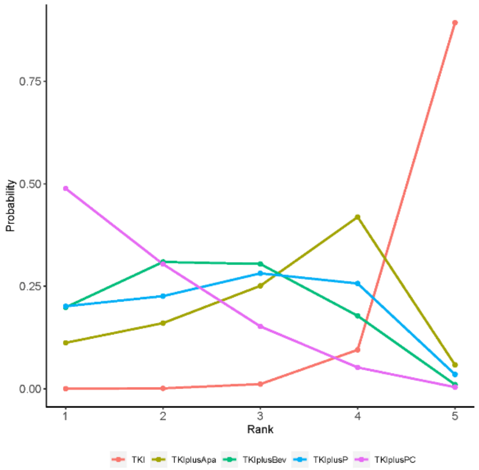

G. (a). Network diagram of comparisons on progression-free survival in patients with non-brain metastasis; (b). Rank profile of comparisons on progression-free survival in patients with non-brain metastasis.

H

a

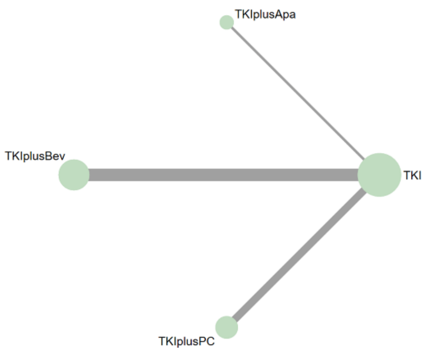

b

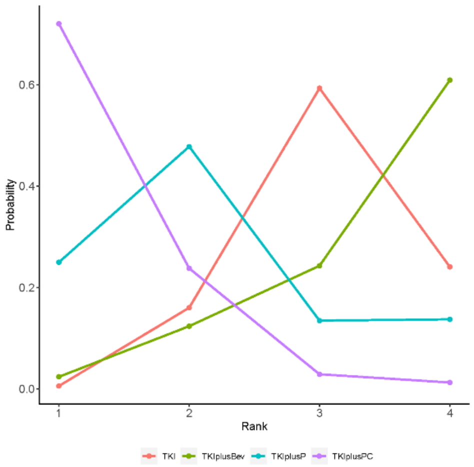

H. (a). Network diagram of comparisons on overall survival in patients with non-brain metastasis; (b). Rank profile of comparisons on overall survival in patients with non-brain metastasis.

I

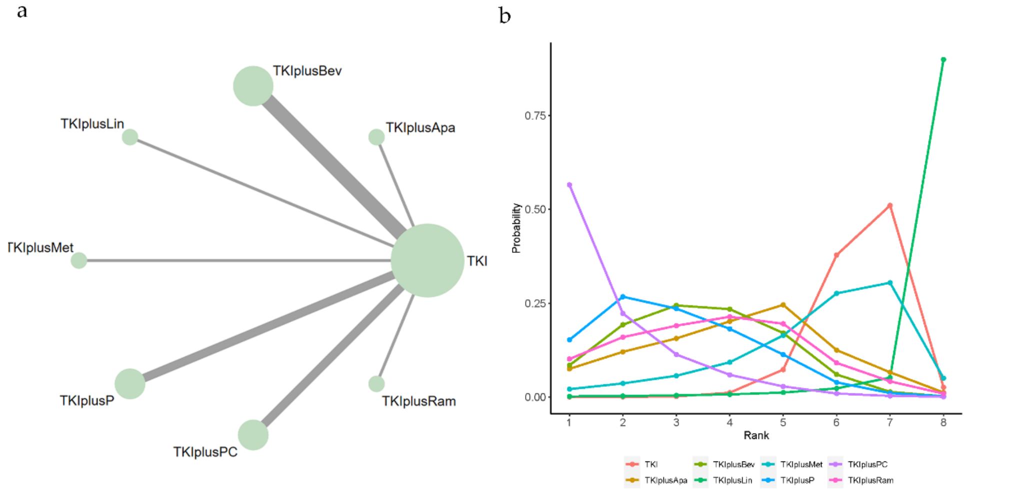

I. (a). Network diagram of comparisons on progression-free survival in non-smokers;(b). Rank profile of comparisons on progression-free survival in non-smokers.

J

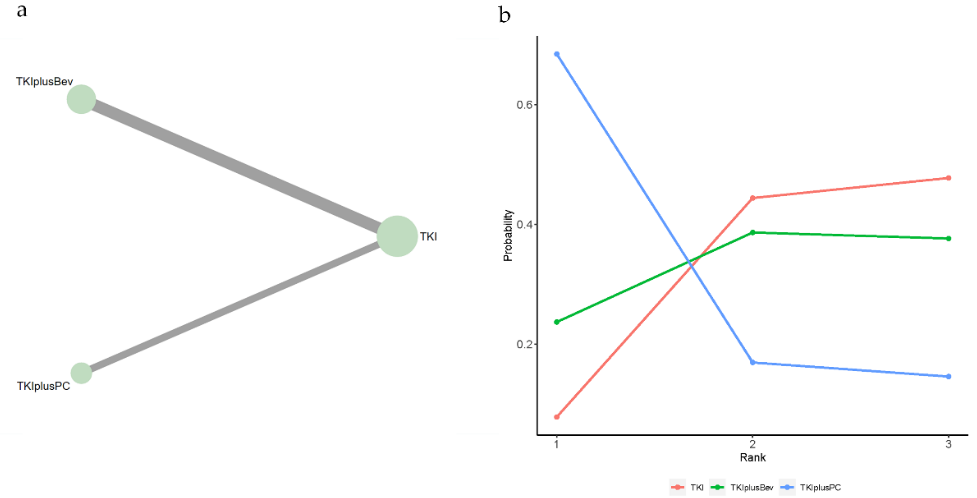

J. (a). Network diagram of comparisons on overall survival in non-smokers; (b). Rank profile of comparisons on overall survival in non-smokers.

K

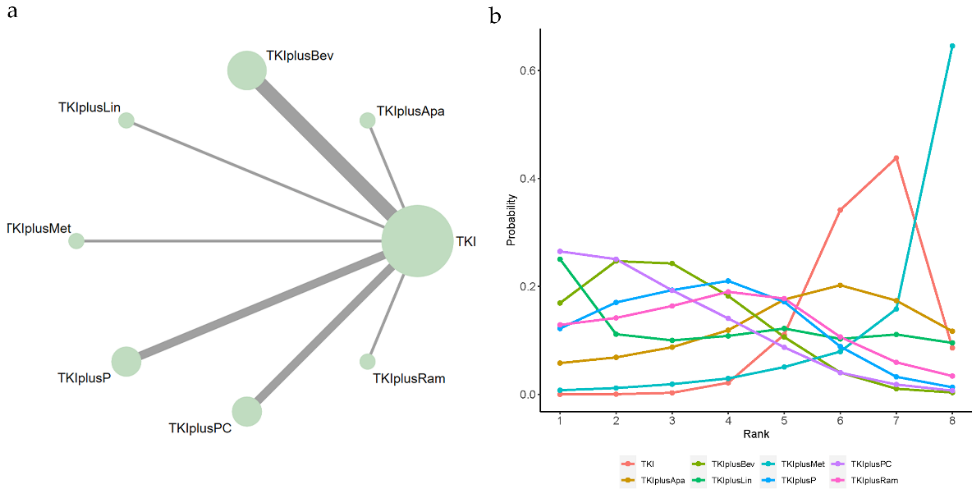

K. (a). Network diagram of comparisons on progression-free survival in smokers; (b). Rank profile of comparisons on progression-free survival in smokers.

L

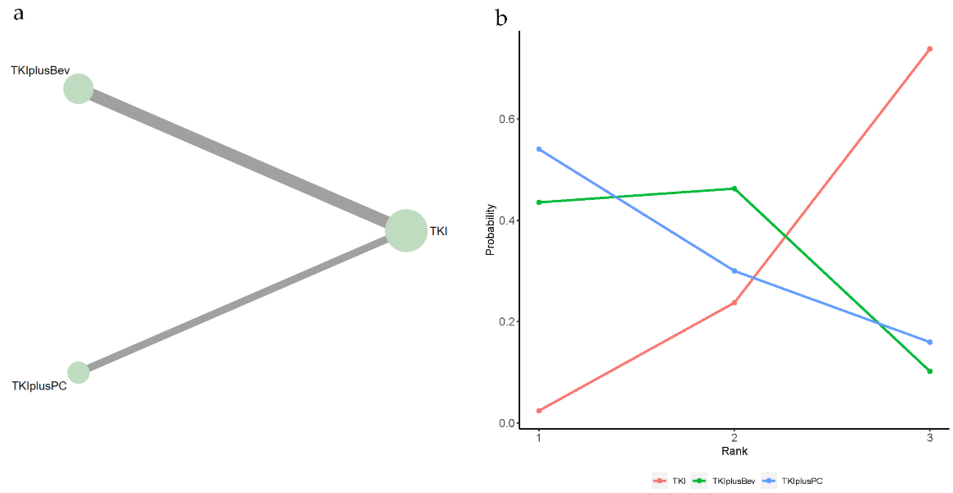

L. (a). Network diagram of comparisons on overall survival in smokers; (b). Rank profile of comparisons on overall survival in smokers.

M

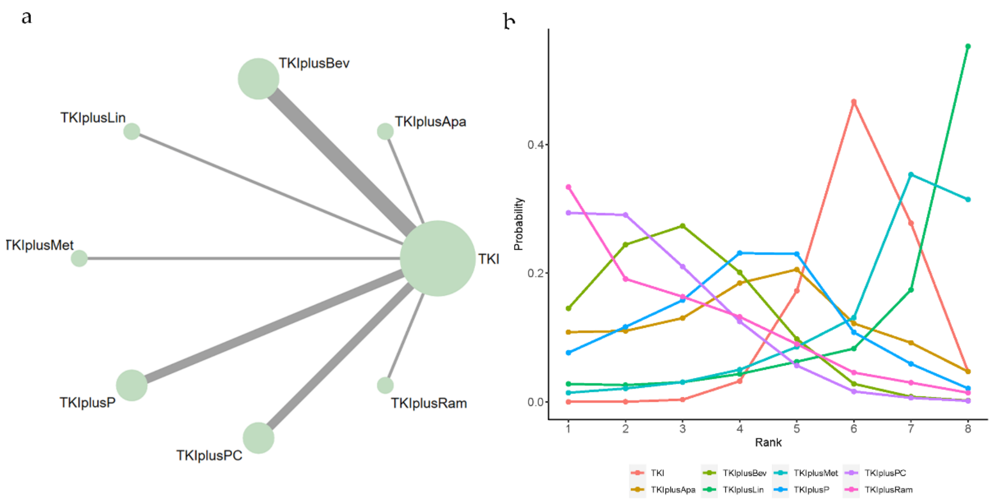

M. (a). Network diagram of comparisons on progression-free survival in male patients; (b). Rank profile of comparisons on progression-free survival in male patients.

N

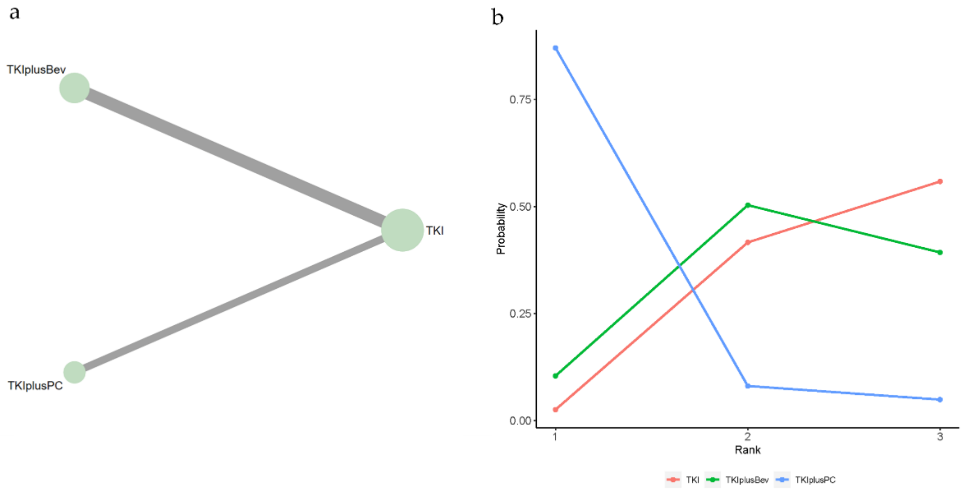

N. (a). Network diagram of comparisons on overall survival in male patients; (b). Rank profile of comparisons on overall survival in male patients.

O

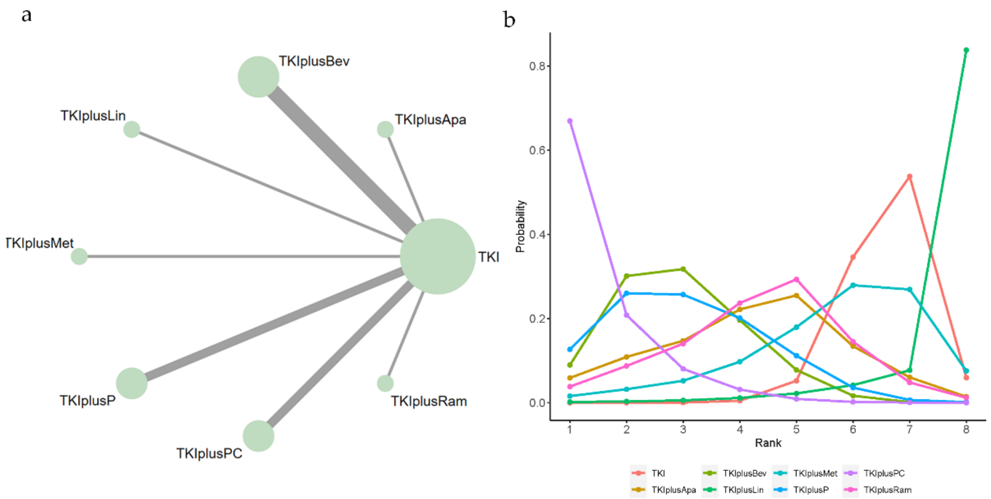

O. (a). Network diagram of comparisons on progression-free survival in female patients; (b). Rank profile of comparisons on progression-free survival in female patients.

P

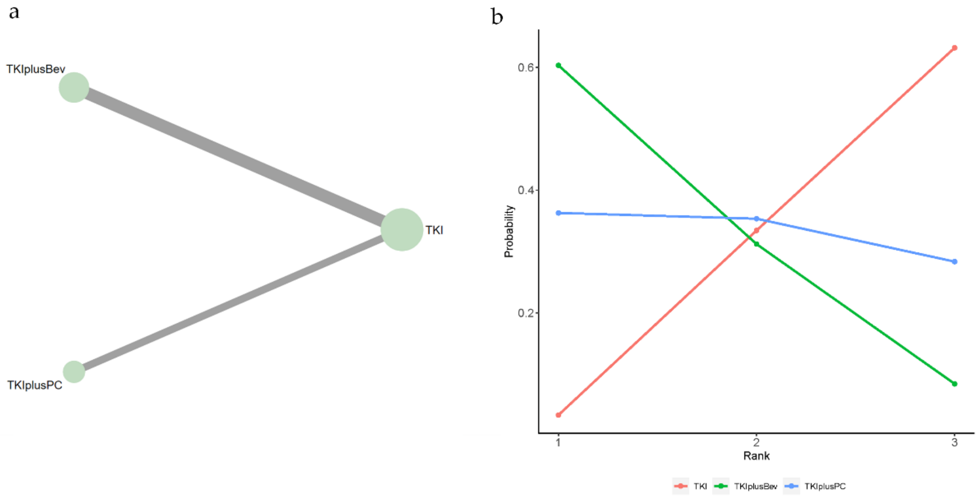

P. (a). Network diagram of comparisons on overall survival in female patients; (b). Rank profile of comparisons on overall survival in female patients.

TKI=Tyrosine kinase inhibitors (TKI) monotherapy; TKIplusP=TKI plus pemetrexed; TKIplusPC=TKI plus pemetrexed & carboplatin; TKIplusBev=TKI plus bevacizumab; TKIplusRam=TKI plus ramucirumab; TKIplusApa=TKI plus apatinib; TKIplusLin=TKI plus linsitinib; TKIplusMet=TKI plus metformin.

A

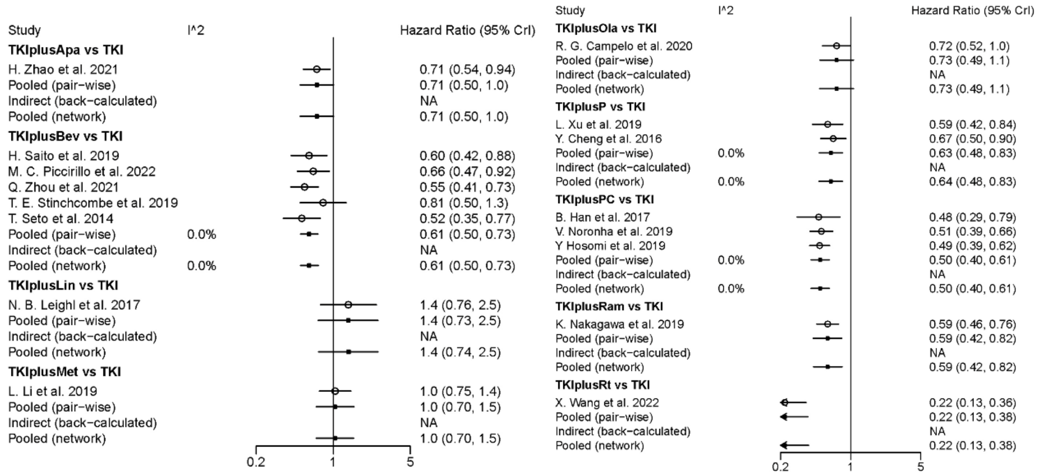
Study
I^2
Hazard Ratio (95% CrI)
TKIplusApa vs TKI
H. Zhao et al. 2021 0.71 (0.54, 0.94)
Pooled (pair-wise) 0.71 (0.50, 1.0)
Indirect (back-calculated) NA
Pooled (network) 0.71 (0.50, 1.0)
TKIplusBev vs TKI
H. Saito et al. 2019 0.60 (0.42, 0.88)
M. C. Piccirillo et al. 2022 0.66 (0.47, 0.92)
Q. Zhou et al. 2021 0.55 (0.41, 0.73)
T. E. Stinchcombe et al. 2019 0.81 (0.50, 1.3)
T. Seto et al. 2014 0.52 (0.35, 0.77)
Pooled (pair-wise) 0.0% 0.61 (0.50, 0.73)
Indirect (back-calculated) NA
Pooled (network) 0.0% 0.61 (0.50, 0.73)
TKIplusLin vs TKI
N. B. Leighl et al. 2017 1.4 (0.76, 2.5)
Pooled (pair-wise) 1.4 (0.73, 2.5)
Indirect (back-calculated) NA
Pooled (network) 1.4 (0.74, 2.5)
TKIplusMet vs TKI
L. Li et al. 2019 1.0 (0.75, 1.4)
Pooled (pair-wise) 1.0 (0.70, 1.5)
Indirect (back-calculated) NA
Pooled (network) 1.0 (0.70, 1.5)
0.2 1 5
Study
I^2
Hazard Ratio (95% CrI)
TKIplusOla vs TKI
R. G. Campelo et al. 2020 0.72 (0.52, 1.0)
Pooled (pair-wise) 0.73 (0.49, 1.1)
Indirect (back-calculated) NA
Pooled (network) 0.73 (0.49, 1.1)
TKIplusP vs TKI
L. Xu et al. 2019 0.59 (0.42, 0.84)
Y. Cheng et al. 2016 0.67 (0.50, 0.90)
Pooled (pair-wise) 0.0% 0.63 (0.48, 0.83)
Indirect (back-calculated) NA
Pooled (network) 0.0% 0.64 (0.48, 0.83)
TKIplusPC vs TKI
B. Han et al. 2017 0.48 (0.29, 0.79)
V. Noronha et al. 2019 0.51 (0.39, 0.66)
Y Hosomi et al. 2019 0.49 (0.39, 0.62)
Pooled (pair-wise) 0.0% 0.50 (0.40, 0.61)
Indirect (back-calculated) NA
Pooled (network) 0.0% 0.50 (0.40, 0.61)
TKIplusRam vs TKI
K. Nakagawa et al. 2019 0.59 (0.46, 0.76)
Pooled (pair-wise) 0.59 (0.42, 0.82)
Indirect (back-calculated) NA
Pooled (network) 0.59 (0.42, 0.82)
TKIplusRt vs TKI
X. Wang et al. 2022 0.22 (0.13, 0.36)
Pooled (pair-wise) 0.22 (0.13, 0.38)
Indirect (back-calculated) NA
Pooled (network) 0.22 (0.13, 0.38)
0.2 1 5

B

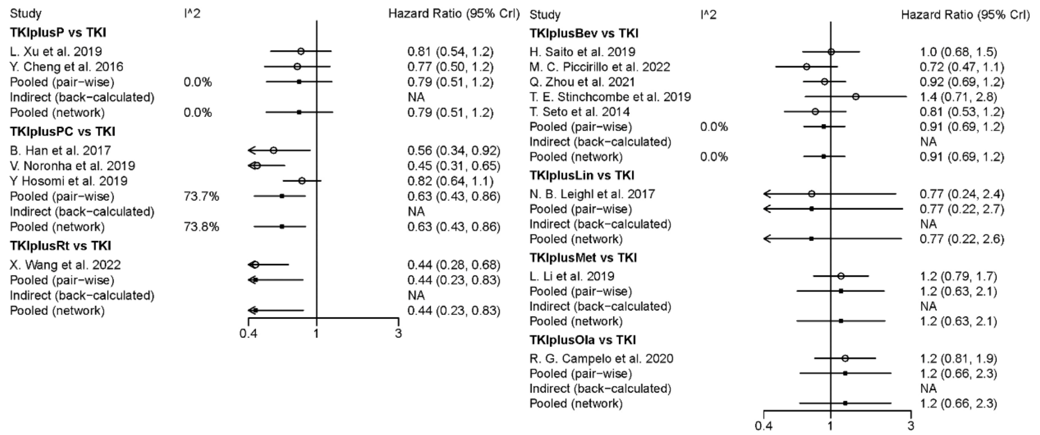
Study
I^2
Hazard Ratio (95% CrI)
TKIplusP vs TKI
L. Xu et al. 2019 0.81 (0.54, 1.2)
Y. Cheng et al. 2016 0.77 (0.50, 1.2)
Pooled (pair-wise) 0.0% 0.79 (0.51, 1.2)
Indirect (back-calculated) NA
Pooled (network) 0.0% 0.79 (0.51, 1.2)
TKIplusPC vs TKI
B. Han et al. 2017 0.56 (0.34, 0.92)
V. Noronha et al. 2019 0.45 (0.31, 0.65)
Y Hosomi et al. 2019 0.82 (0.64, 1.1)
Pooled (pair-wise) 73.7% 0.63 (0.43, 0.86)
Indirect (back-calculated) NA
Pooled (network) 73.8% 0.63 (0.43, 0.86)
TKIplusRt vs TKI
X. Wang et al. 2022 0.44 (0.28, 0.68)
Pooled (pair-wise) 0.44 (0.23, 0.83)
Indirect (back-calculated) NA
Pooled (network) 0.44 (0.23, 0.83)
0.4 1 3
Study
I^2
Hazard Ratio (95% CrI)
TKIplusBev vs TKI
H. Saito et al. 2019 1.0 (0.68, 1.5)
M. C. Piccirillo et al. 2022 0.72 (0.47, 1.1)
Q. Zhou et al. 2021 0.92 (0.69, 1.2)
T. E. Stinchcombe et al. 2019 1.4 (0.71, 2.8)
T. Seto et al. 2014 0.81 (0.53, 1.2)
Pooled (pair-wise) 0.0% 0.91 (0.69, 1.2)
Indirect (back-calculated) NA
Pooled (network) 0.0% 0.91 (0.69, 1.2)
TKIplusLin vs TKI
N. B. Leighl et al. 2017 0.77 (0.24, 2.4)
Pooled (pair-wise) 0.77 (0.22, 2.7)
Indirect (back-calculated) NA
Pooled (network) 0.77 (0.22, 2.6)
TKIplusMet vs TKI
L. Li et al. 2019 1.2 (0.79, 1.7)
Pooled (pair-wise) 1.2 (0.63, 2.1)
Indirect (back-calculated) NA
Pooled (network) 1.2 (0.63, 2.1)
TKIplusOla vs TKI
R. G. Campelo et al. 2020 1.2 (0.81, 1.9)
Pooled (pair-wise) 1.2 (0.66, 2.3)
Indirect (back-calculated) NA
Pooled (network) 1.2 (0.66, 2.3)
0.4 1 3

C

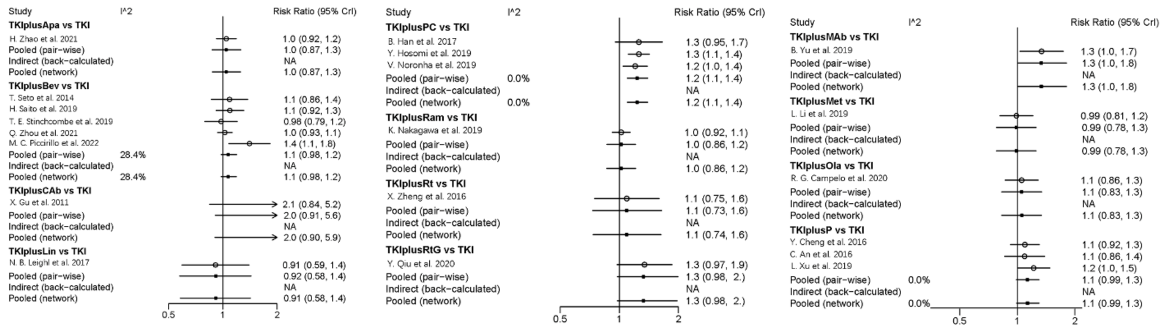
Study
I^2
Risk Ratio (95% CrI)
TKIplusApa vs TKI
H. Zhao et al. 2021 1.0 (0.92, 1.2)
Pooled (pair-wise) 1.0 (0.87, 1.3)
Indirect (back-calculated) NA
Pooled (network) 1.0 (0.87, 1.3)
TKIplusBev vs TKI
T. Seto et al. 2014 1.1 (0.86, 1.4)
H. Saito et al. 2019 1.1 (0.92, 1.3)
T. E. Stinchcombe et al. 2019 0.98 (0.79, 1.2)
Q. Zhou et al. 2021 1.0 (0.93, 1.1)
M. C. Piccirillo et al. 2022 1.4 (1.1, 1.8)
Pooled (pair-wise) 28.4% 1.1 (0.98, 1.2)
Indirect (back-calculated) NA
Pooled (network) 28.4% 1.1 (0.98, 1.2)
TKIplusCAb vs TKI
X. Gu et al. 2011 2.1 (0.84, 5.2)
Pooled (pair-wise) 2.0 (0.91, 5.6)
Indirect (back-calculated) NA
Pooled (network) 2.0 (0.90, 5.9)
TKIplusLin vs TKI
N. B. Leighl et al. 2017 0.91 (0.59, 1.4)
Pooled (pair-wise) 0.92 (0.58, 1.4)
Indirect (back-calculated) NA
Pooled (network) 0.91 (0.58, 1.4)
0.5 1 2
Study
I^2
Risk Ratio (95% CrI)
TKIplusPC vs TKI
B. Han et al. 2017 1.3 (0.95, 1.7)
Y. Hosomi et al. 2019 1.3 (1.1, 1.4)
V. Noronha et al. 2019 1.2 (1.0, 1.4)
Pooled (pair-wise) 0.0% 1.2 (1.1, 1.4)
Indirect (back-calculated) NA
Pooled (network) 0.0% 1.2 (1.1, 1.4)
TKIplusRam vs TKI
K. Nakagawa et al. 2019 1.0 (0.92, 1.1)
Pooled (pair-wise) 1.0 (0.86, 1.2)
Indirect (back-calculated) NA
Pooled (network) 1.0 (0.86, 1.2)
TKIplusRt vs TKI
X. Zheng et al. 2016 1.1 (0.75, 1.6)
Pooled (pair-wise) 1.1 (0.73, 1.6)
Indirect (back-calculated) NA
Pooled (network) 1.1 (0.74, 1.6)
TKIplusRtG vs TKI
Y. Qiu et al. 2020 1.3 (0.97, 1.9)
Pooled (pair-wise) 1.3 (0.98, 2.)
Indirect (back-calculated) NA
Pooled (network) 1.3 (0.98, 2.)
0.5 1 2
Study
I^2
Risk Ratio (95% CrI)
TKIplusMAb vs TKI
B. Yu et al. 2019 1.3 (1.0, 1.7)
Pooled (pair-wise) 1.3 (1.0, 1.8)
Indirect (back-calculated) NA
Pooled (network) 1.3 (1.0, 1.8)
TKIplusMet vs TKI
L. Li et al. 2019 0.99 (0.81, 1.2)
Pooled (pair-wise) 0.99 (0.78, 1.3)
Indirect (back-calculated) NA
Pooled (network) 0.99 (0.78, 1.3)
TKIplusOla vs TKI
R. G. Campelo et al. 2020 1.1 (0.86, 1.3)
Pooled (pair-wise) 1.1 (0.83, 1.3)
Indirect (back-calculated) NA
Pooled (network) 1.1 (0.83, 1.3)
TKIplusP vs TKI
Y. Cheng et al. 2016 1.1 (0.92, 1.3)
C. An et al. 2016 1.1 (0.86, 1.4)
L. Xu et al. 2019 1.2 (1.0, 1.5)
Pooled (pair-wise) 0.0% 1.1 (0.99, 1.3)
Indirect (back-calculated) NA
Pooled (network) 0.0% 1.1 (0.99, 1.3)
0.5 1 2

D

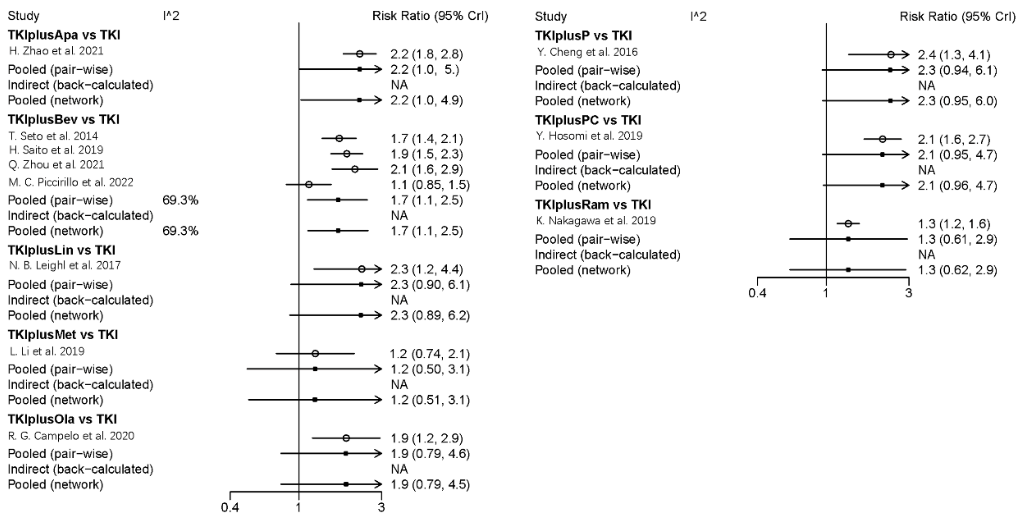

Figure S7. Heterogeneity assessing of the studies enrolled. (A) Studies for analysis on progression free survival (PFS). (B) Studies for analysis on overall survival (OS). (C) Studies for analysis on objective response rate (ORR). (D) Studies for analysis on adverse events of grade 3 or higher (≥Grade 3 AEs). TKI=Tyrosine kinase inhibitors (TKI) monotherapy, TKIplusP=TKI plus pemetrexed, TKIplusPC=TKI plus pemetrexed & carboplatin, TKIplusBev=TKI plus bevacizumab, TKIplusRam=TKI plus ramucirumab, TKIplusApa=TKI plus apatinib, TKIplusCAb=TKI plus cryoablation, TKIplusMAb=TKI plus microwave ablation, TKIplusLin=TKI plus linsitinib, TKIplusMet=TKI plus metformin, TKIplusOla=TKI plus olaparib, TKIplusRt=TKI plus radiation, TKIplusRtG=TKI plus radiotherapy & GM-CSF.

A

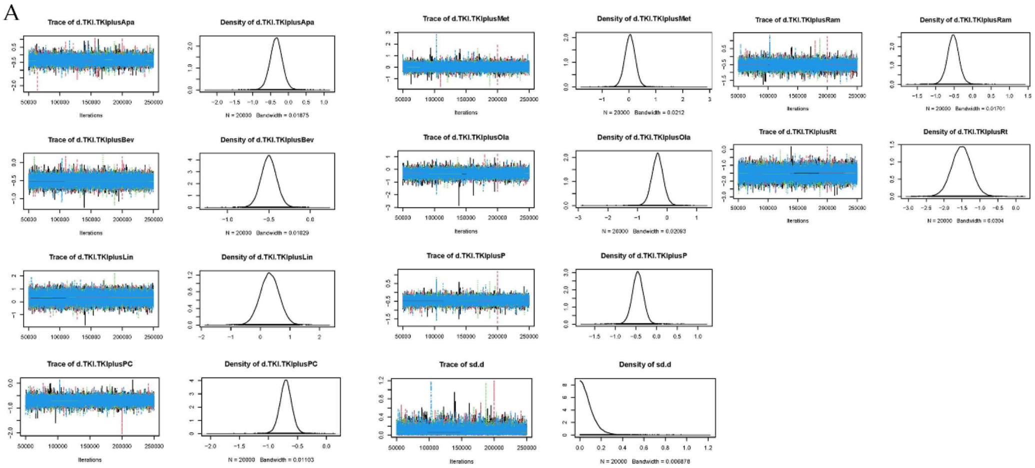
Trace of d.TKI.TKIplusApa
Density of d.TKI.TKIplusApa
Trace of d.TKI.TKIplusMet
Density of d.TKI.TKIplusMet
Trace of d.TKI.TKIplusRam
Density of d.TKI.TKIplusRam
Trace of d.TKI.TKIplusBev
Density of d.TKI.TKIplusBev
Trace of d.TKI.TKIplusOla
Density of d.TKI.TKIplusOla
Trace of d.TKI.TKIplusRt
Density of d.TKI.TKIplusRt
Trace of d.TKI.TKIplusLin
Density of d.TKI.TKIplusLin
Trace of d.TKI.TKIplusP
Density of d.TKI.TKIplusP
Trace of d.TKI.TKIplusPC
Density of d.TKI.TKIplusPC
Trace of sd.d
Density of sd.d

B

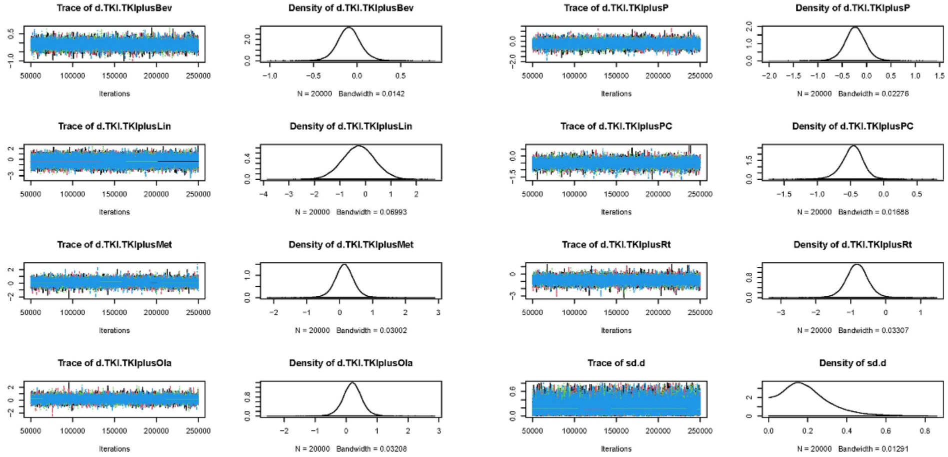
Trace of d.TKI.TKIplusBev
Density of d.TKI.TKIplusBev
N = 20000 Bandwidth = 0.0142
Trace of d.TKI.TKIplusP
Density of d.TKI.TKIplusP
N = 20000 Bandwidth = 0.02276
Trace of d.TKI.TKIplusLin
Density of d.TKI.TKIplusLin
N = 20000 Bandwidth = 0.06993
Trace of d.TKI.TKIplusPC
Density of d.TKI.TKIplusPC
N = 20000 Bandwidth = 0.01688
Trace of d.TKI.TKIplusMet
Density of d.TKI.TKIplusMet
N = 20000 Bandwidth = 0.03002
Trace of d.TKI.TKIplusRt
Density of d.TKI.TKIplusRt
N = 20000 Bandwidth = 0.03307
Trace of d.TKI.TKIplusOla
Density of d.TKI.TKIplusOla
N = 20000 Bandwidth = 0.03208
Trace of sd.d
Density of sd.d
N = 20000 Bandwidth = 0.01291

C

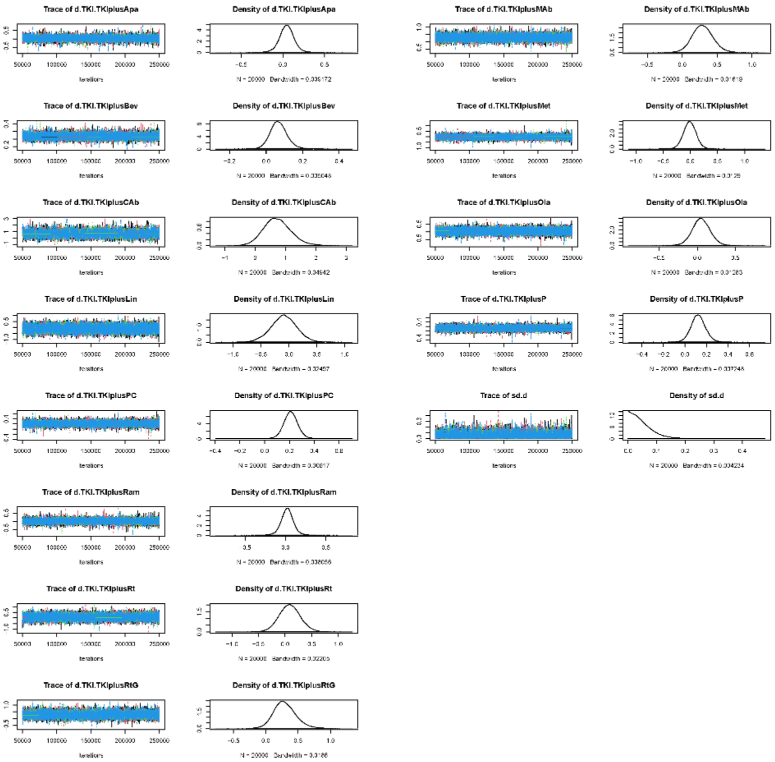
Trace of d.TKI.TKIplusApa
Density of d.TKI.TKIplusApa
Trace of d.TKI.TKIplusMAb
Density of d.TKI.TKIplusMAb
Trace of d.TKI.TKIplusBev
Density of d.TKI.TKIplusBev
Trace of d.TKI.TKIplusMet
Density of d.TKI.TKIplusMet
Trace of d.TKI.TKIplusCAb
Density of d.TKI.TKIplusCAb
Trace of d.TKI.TKIplusOla
Density of d.TKI.TKIplusOla
Trace of d.TKI.TKIplusLin
Density of d.TKI.TKIplusLin
Trace of d.TKI.TKIplusP
Density of d.TKI.TKIplusP
Trace of d.TKI.TKIplusPC
Density of d.TKI.TKIplusPC
Trace of sd.d
Density of sd.d
Trace of d.TKI.TKIplusRam
Density of d.TKI.TKIplusRam
Trace of d.TKI.TKIplusRt
Density of d.TKI.TKIplusRt
Trace of d.TKI.TKIplusRtG
Density of d.TKI.TKIplusRtG

D

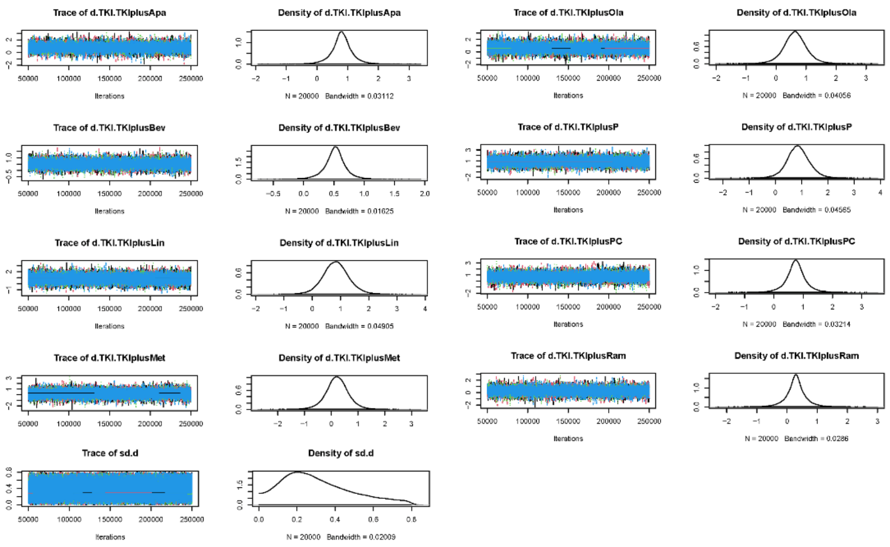

Figure S8. Trace plots and density plots.

(A) Progression free survival (PFS) network model, (B) overall survival (OS) network model, (C) objective response rate (ORR) network model, (D) adverse events of grade 3 or higher (≥Grade 3 AEs) network model. TKI=Tyrosine kinase inhibitors (TKI) monotherapy; TKIplusP=TKI plus pemetrexed; TKIplusPC=TKI plus pemetrexed & carboplatin; TKIplusBev=TKI plus bevacizumab; TKIplusRam=TKI plus ramucirumab; TKIplusApa=TKI plus apatinib; TKIplusCAb=TKI plus cryoablation; TKIplusMAb=TKI plus microwave ablation; TKIplusLin=TKI plus linsitinib; TKIplusMet=TKI plus metformin; TKIplusOla=TKI plus olaparib; TKIplusRt=TKI plus radiation; TKIplusRtG=TKI plus radiotherapy & GM-CSF.

A

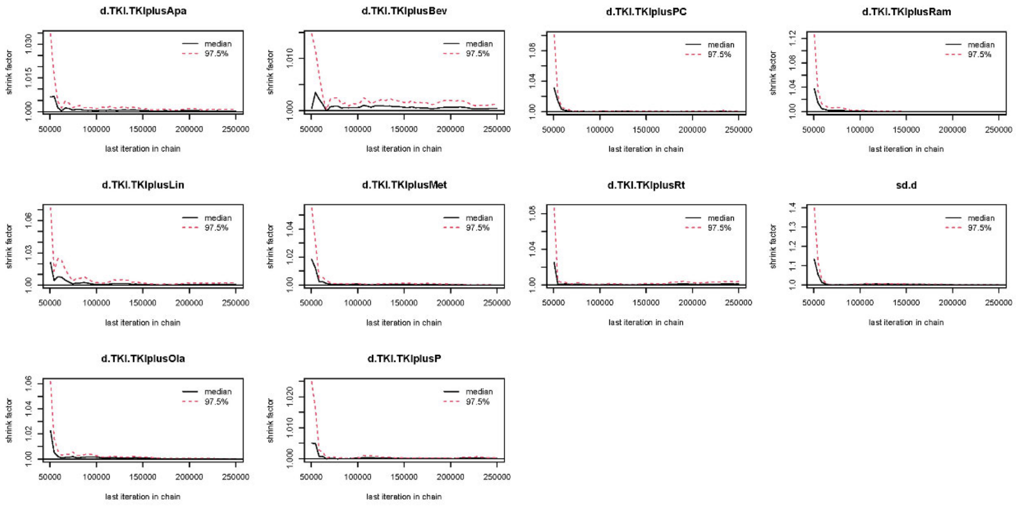
d.TKI.TKIplusApa
d.TKI.TKIplusBev
d.TKI.TKIplusPC
d.TKI.TKIplusRam
d.TKI.TKIplusLin
d.TKI.TKIplusMet
d.TKI.TKIplusRt
sd.d
d.TKI.TKIplusOla
d.TKI.TKIplusP
median
97.5%
shrink factor
last iteration in chain

B

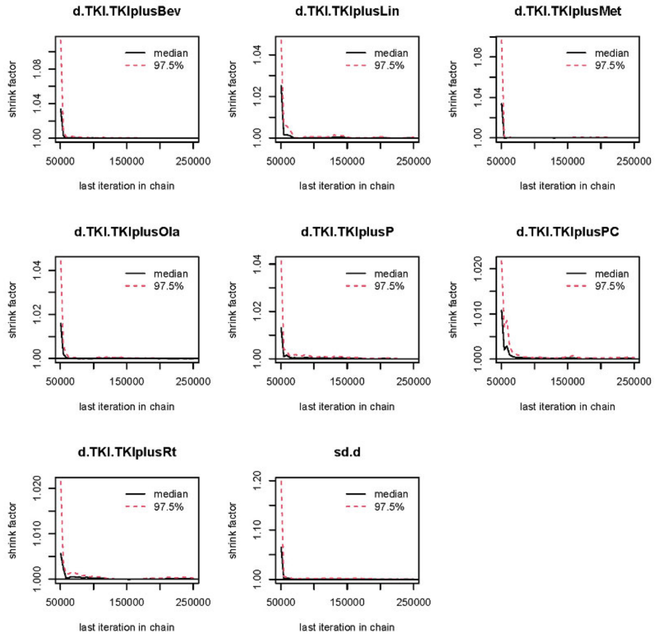
d.TKI.TKIplusBev
d.TKI.TKIplusLin
d.TKI.TKIplusMet
d.TKI.TKIplusOla
d.TKI.TKIplusP
d.TKI.TKIplusPC
d.TKI.TKIplusRt
sd.d
median
97.5%
shrink factor
last iteration in chain

C

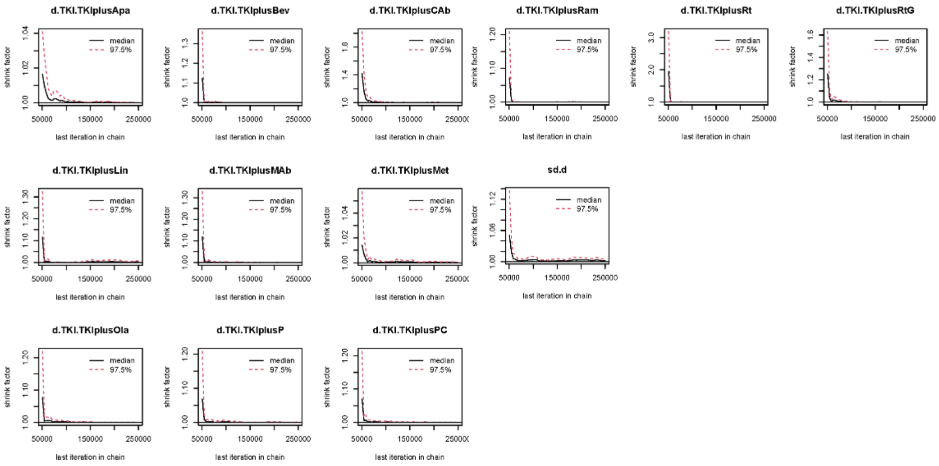

D

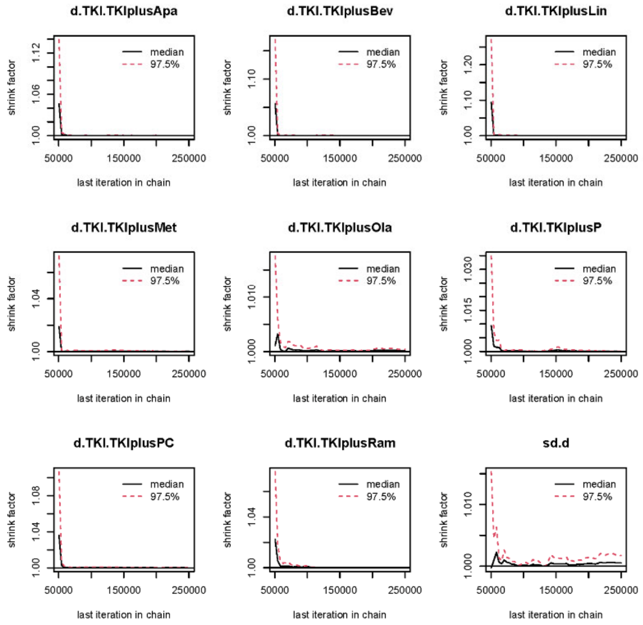

Figure S9. Brooks-Gelman-Rubin plots.

(A) Progression free survival (PFS) network model, (B) overall survival (OS) network model, (C) objective response rate (ORR) network model, (D) adverse events of grade 3 or higher (≥Grade 3 AEs) network model. TKI=Tyrosine kinase inhibitors (TKI) monotherapy; TKIplusP=TKI plus pemetrexed; TKIplusPC=TKI plus pemetrexed & carboplatin; TKIplusBev=TKI plus bevacizumab; TKIplusRam=TKI plus ramucirumab; TKIplusApa=TKI plus apatinib; TKIplusCAb=TKI plus cryoablation; TKIplusMAb=TKI plus microwave ablation; TKIplusLin=TKI plus linsitinib; TKIplusMet=TKI plus metformin; TKIplusOla=TKI plus olaparib; TKIplusRt=TKI plus radiation; TKIplusRtG=TKI plus radiotherapy & GM-CSF.

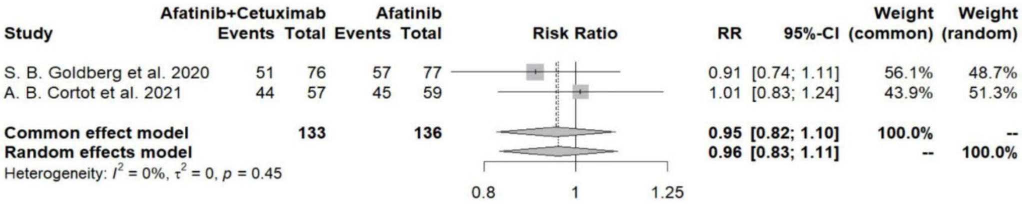

Figure S10. Forest plots of objective response rate (ORR) of afatinib plus cetuximab compared with that of afatinib monotherapy.
